# Friction-Induced Thermal Effects in an FGM Layer in Contact with a Homogeneous Layer

**DOI:** 10.3390/ma19071299

**Published:** 2026-03-25

**Authors:** Katarzyna Topczewska

**Affiliations:** Faculty of Mechanical Engineering, Bialystok University of Technology, 45C Wiejska Street, 15-351 Bialystok, Poland; k.topczewska@pb.edu.pl

**Keywords:** frictional heat, temperature, layer, functionally gradient material, convective heat transfer, contact conductance

## Abstract

An analytical model of frictional heat transfer during the uniform sliding of two layers is proposed. One layer is composed of a functionally graded material (FGM) with a thermal conductivity coefficient that varies exponentially across its thickness, while the second layer is homogeneous, with constant thermophysical properties. The thermal problem of friction is formulated as an initial boundary value problem of heat conduction, accounting for the thermal contact conductance and convective heat exchange with the environment. An exact solution for constant friction power was obtained using the Laplace integral transform, supplemented by an asymptotic form for the initial stage of heating. Based on these analytical solutions, a comprehensive study was carried out for a frictional system comprising a ceramic–metal FGM composite in contact with a homogeneous friction material. A dimensional analysis allowed for both a qualitative and quantitative investigation into the influence of contact conductance, convective heat exchange, layer thickness and the FGM gradient parameter on the temperature evolution and distribution, as well as the time to reach the steady state. It was demonstrated that the implementation of an appropriately graded material can substantially improve thermal operating conditions by enhancing heat dissipation into the material bulk and intensifying convective cooling.

## 1. Introduction

Initial boundary value problems of heat conduction incorporating heat generation due to friction (so-called frictional heat problems) in a two-layer system play a crucial role in the development of analytical models of frictional heating processes when the effective depth of heat penetration exceeds the thickness of the elements of the friction pair [1,2,3]. An exact solution to this problem, together with the history of its development for a system of two sliding layers made of homogeneous materials, was presented in [4]. In the problem formulation, convective cooling on the free surfaces of the layers was taken into account, as well as the imperfect contact of the frictional surfaces, resulting from the thermal resistance caused by the roughness of the contacting surfaces. The corresponding problem for layers made of functionally graded materials (FGMs), possessing a thermal conductivity coefficient that varies exponentially through thickness, was analyzed in [5]. At first glance, it might seem that the solution for homogeneous materials [4] could be obtained by taking the limiting case of vanishing FGM gradient parameters in the solution presented in [5]. Unfortunately, this turns out to be very technically complicated, since in the resulting solution, the gradient parameter appears in the denominator of the arguments of Bessel functions. Consequently, it would be necessary to perform a labor-intensive procedure of replacing the Bessel functions with their asymptotic representations for large argument values. In order to avoid this difficulty, the frictional heat problem for a system consisting of an FGM layer sliding uniformly over the surface of a homogeneous layer is considered in this article.

Analytical models of frictional heating in systems in which one component is made of a functionally graded material (FGM) and the other of a homogeneous material have been the subject of previous studies. The spatial–temporal distributions of the temperature field in a system of two sliding half-spaces (FGM and homogeneous), assuming perfect thermal contact at the friction interface, were analyzed in [6]. In turn, ref. [7] proposed an analytical model of frictional heat generation in a system consisting of an FGM layer and a homogeneous half-space. This model accounts for convective cooling on the free surface of the FGM layer, assuming imperfect thermal contact conditions between the layer and the half-space.

An FGM layer is not only used as an independent component in analytical models of frictional heating processes, but it may also serve as a protective thermal barrier coating (TBC) deposited on a homogeneous substrate. A mathematical model for determining the temperature generated as a result of heating the surface of an FGM layer, deposited on a homogeneous substrate and subjected to a heat flux with a temporal profile applied a priori, was proposed in [8]. In contrast, ref. [9] presented an exact solution to the frictional heat problem for a system of two sliding half-spaces; one was made of a homogeneous material, while the surface of the other was coated with a protective FGM layer.

The aim of the present study was to obtain an exact solution to the frictional heat problem for two layers, one made of a functionally graded material (FGM) and the other of a homogeneous material. A model was proposed accounting for thermal contact conductance (thermal resistance at the friction interface) as well as convective heat exchange with the surrounding environment on the free surfaces of the layers. The obtained solution was verified by checking its compliance with the boundary conditions and the initial condition. An appropriate asymptotic solution for the initial stage of the frictional heating process was also presented.

## 2. The Statement of the Problem

**Figure 1:**
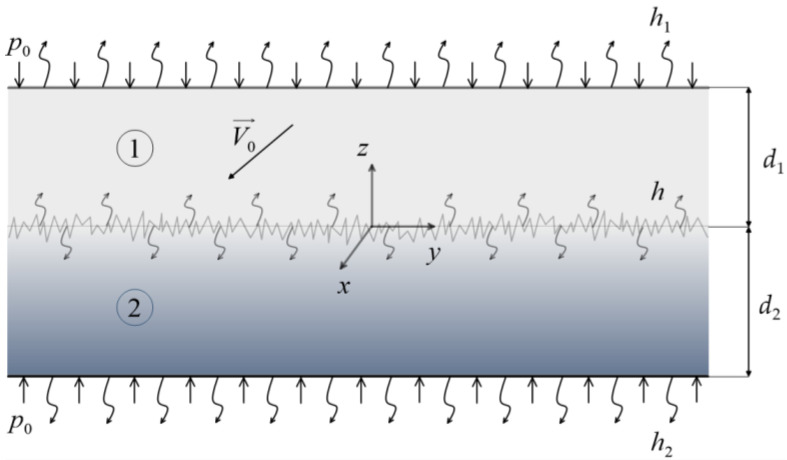
A schematic of the two-layer system.

With respect to a Cartesian coordinate system Oxyz, a two-layer system is considered. The first layer (of thickness $d_{1}$) is made of a homogeneous material, whereas the second layer (of thickness $d_{2}$) consists of a functionally graded material (FGM) with a thermal conductivity coefficient varying along its thickness (Figure 1). The layers are subjected to a uniformly distributed normal compressive pressure $p_{0}$, acting on their external surfaces $z=d_{1}$ and $z=-d_{2}$. Subsequently, at the initial time t=0, the layers begin to slide relative to each other with a constant velocity $V_{0}$ in the direction of the *x*-axis. As a result of friction at the contact surfaces $z=0^{+}$ and $z=0^{-}$, heat is generated and conducted into the elements of the friction pair.

The proposed mathematical model of the frictional heat transfer is based on the following assumptions:

1. The heat generated is absorbed by the layers in the direction normal to the contact surface (along the z-axis).
2. The thermal contact between the layers is imperfect, i.e., the sum of the heat fluxes directed into each layer equals the specific friction power $q_{0}=fp_{0}V_{0}$ (*f*–coefficient of friction), while their difference is proportional to the temperature jump between the frictional surfaces of the layers. The coefficient of proportionality in this relation is the thermal contact conductivity h>0.
3. The external surfaces of the layers are convectively cooled with heat transfer coefficients $h_{l}\geq 0$, l=1, 2.
4. The thermal conductivity of the functionally graded layer increases exponentially with distance from the friction surface.
5. The thermophysical properties of the materials are assumed to be independent of temperature.

It should be noted that the application of the model has some limitations resulting from the simplifying assumptions made. The thermal sensitivity of materials was neglected, and an exponential function was used to describe changes in thermal conductivity in the FGM layer in order to obtain an analytical solution. However, this naturally limits the applicability of the model to FGMs with such features. In reality, the distribution of FGM properties depends on the material manufacturing process and will not necessarily follow an exponential or any other specific functional dependence [10]. Therefore, some level of curve fitting has to be applied. The exponential function provides a convenient representation of a smooth transition between the properties of the FGM components. Moreover, the exponential decay rate (gradient parameter of FGM) can be adjusted to improve the fit of dependency.

On the basis of the above assumptions, to determine the spatial–temporal distribution of temperature T(z,t) within the layers, the following frictional heat transfer problem is formulated:

(1) $$K_{1}\frac{\partial^{2}T(z,t)}{\partial z^{2}}=\rho_{1}C_{1}\frac{\partial T(z,t)}{\partial t},\;0<z<d_{1},\;t>0,$$

(2) $$\frac{\partial }{\partial z}\left[K_{2}(z)\frac{\partial T(z,t)}{\partial z}\right]=\rho_{2}C_{2}\frac{\partial T(z,t)}{\partial t},-d_{2}<z<0,\;t>0,$$

(3) $${\left.{\left.K_{2}(z)\frac{\partial T(z,t)}{\partial z}\right|}_{z=0^{-}}-K_{1}\frac{\partial T(z,t)}{\partial z}\right|}_{z=0^{+}}=q_{0},\;t>0,$$

(4) $${\left.{\left.K_{2}(z)\frac{\partial T(z,t)}{\partial z}\right|}_{z=0^{-}}+K_{1}\frac{\partial T(z,t)}{\partial z}\right|}_{z=0^{+}}=h\;[T(0^{+},t)-T(0^{-},t)],\;t>0,$$

(5) $${\left.K_{1}\frac{\partial T(z,t)}{\partial z}\right|}_{z=d_{1}}=h_{1}[T_{0}-T(d_{1},t)],\;t>0,$$

(6) $${\left.K_{2}(z)\frac{\partial T(z,t)}{\partial z}\right|}_{z=-d_{2}}=h_{2}[T(-d_{2},t)-T_{0}],\;t>0,$$

(7) $$T(z,0)=T_{0},-d_{2}\leq z\leq d_{1},$$

where [11,12]

(8) $$K_{2}(z)=K_{2,1}e^{-\gamma^{*}\frac{z}{d_{2}}},\;\gamma^{*}=\ln\left(\frac{K_{2,2}}{K_{2,1}}\right),\;K_{2}(0)=K_{2,1},\;K_{2}(-d_{2})=K_{2,2},$$

$K_{2,m}$—thermal conductivity of the FGM components m=1, 2; $\gamma^{*}$—gradient parameter of the FGM layer; $K_{l}$—thermal conductivity of the upper (l=1) and lower (l=2) layer materials; $\rho_{l}$—density; $C_{l}$—specific heat; $T_{0}$—initial temperature of the system. Here and in the following, all values and parameters referring to the upper $0\leq z\leq d_{1}$ or lower $-d_{2}\leq z\leq 0$ layer will be denoted by the subscripts l=1 or l=2, respectively. The limiting values of temperature and heat flux intensity, approached from the positive or negative direction along the *z*-axis, are denoted by $0^{+}$ and $0^{-}$, respectively.

The following dimensionless variables and parameters are introduced:

(9) $$\begin{array}{c} \zeta =\frac{z}{d_{1}},\;\tau =\frac{k_{1}t}{d_{1}^{2}},\;d^{*}=\frac{d_{2}}{d_{1}},\;K^{*}=\frac{K_{2,1}}{K_{1}},\;k^{*}=\frac{k_{2}}{k_{1}},\;Bi=\frac{hd_{1}}{K_{1}},\;Bi_{1}=\frac{h_{1}d_{1}}{K_{1}},\\ \;Bi_{2}=\frac{h_{2}d_{2}}{K_{2,2}},\;\Theta^{*}=\frac{\Theta }{\Theta_{0}}, \end{array}$$

where

(10) $$k_{1}=\frac{K_{1}}{\rho_{1}C_{1}},\;k_{2}=\frac{K_{2,1}}{\rho_{2}C_{2}},\;\Theta_{0}=\frac{q_{0}d_{1}}{K_{1}},$$

$\Theta =T-T_{0}$—the temperature increase resulting from frictional heating. With the use of the definitions introduced (9) and (10), the problem described by (1)–(8) is written in the following dimensionless form:

(11) $$\frac{\partial^{2}\Theta^{*}(\zeta ,\tau )}{\partial \zeta^{2}}=\frac{\partial \Theta^{*}(\zeta ,\tau )}{\partial \tau },\;0<\zeta <1,\;\tau >0,$$

(12) $$\frac{\partial^{2}\Theta^{*}(\zeta ,\tau )}{\partial \zeta^{2}}-\frac{\gamma^{*}}{d^{*}}\frac{\partial \Theta^{*}(\zeta ,\tau )}{\partial \zeta }=\frac{e^{\gamma^{*}\frac{\zeta }{d^{*}}}}{k^{*}}\frac{\partial \Theta^{*}(\zeta ,\tau )}{\partial \tau },-d^{*}<\zeta <0,\;\tau >0,$$

(13) $$K^{*}{\left.\frac{\partial \Theta^{*}(\zeta ,\tau )}{\partial \zeta }\right|}_{\zeta =0^{-}}-{\left.\frac{\partial \Theta^{*}(\zeta ,\tau )}{\partial \zeta }\right|}_{\zeta =0^{+}}=1,\;\tau >0,$$

(14) $$K^{*}{\left.\frac{\partial \Theta^{*}(\zeta ,\tau )}{\partial \zeta }\right|}_{\zeta =0^{-}}+{\left.\frac{\partial \Theta^{*}(\zeta ,\tau )}{\partial \zeta }\right|}_{\zeta =0^{+}}=Bi\;[\Theta^{*}(0^{+},\tau )-\Theta^{*}(0^{-},\tau )],\;\tau >0,$$

(15) $${\left.\frac{\partial \Theta^{*}(\zeta ,\tau )}{\partial \zeta }\right|}_{\zeta =1}=-Bi_{1}\;\Theta^{*}(1,\tau ),\;\tau >0,$$

(16) $$d^{*}{\left.\frac{\partial \Theta^{*}(\zeta ,\tau )}{\partial \zeta }\right|}_{\zeta =-d^{*}}=Bi_{2}\;\Theta^{*}(-d^{*},\tau ),\;\tau >0,$$

(17) $$\Theta^{*}(\zeta ,0)=0,-d^{*}\leq \zeta \leq 1.$$

## 3. The Solution of the Problem in the Laplace Integral Transform Space

We then apply the Laplace integral transform to the initial boundary value heat conduction problem (11)–(17) [13]:

(18) $${\bar{\Theta }}^{*}(\zeta ,p)\equiv L[\Theta^{*}(\zeta ,\tau );p]=\int_{0}^{\infty }\Theta^{*}(\zeta ,\tau )e^{-p\tau }d\tau ,$$

which yields the following boundary value problem for two second-order ordinary differential equations:

(19) $$\frac{d^{2}{\bar{\Theta }}^{*}(\zeta ,p)}{d\zeta^{2}}-p{\bar{\Theta }}^{*}(\zeta ,p)=0,\;0<\zeta <1,$$

(20) $$\frac{d^{2}{\bar{\Theta }}^{*}(\zeta ,p)}{d\zeta^{2}}-\frac{\gamma^{*}}{d^{*}}\frac{d{\bar{\Theta }}^{*}(\zeta ,p)}{d\zeta }-\frac{p}{k^{*}}e^{\gamma^{*}\frac{\zeta }{d^{*}}}{\bar{\Theta }}^{*}(\zeta ,p)=0,-d^{*}<\zeta <0,$$

(21) $${\left.K^{*}\frac{d{\bar{\Theta }}^{*}(\zeta ,p)}{d\zeta }\right|}_{\zeta =0^{-}}-\;{\left.\frac{d{\bar{\Theta }}^{*}(\zeta ,p)}{d\zeta }\right|}_{\zeta =0^{+}}=\frac{1}{p},$$

(22) $${\left.K^{*}\frac{d{\bar{\Theta }}^{*}(\zeta ,p)}{d\zeta }\right|}_{\zeta =0^{-}}+{\left.\frac{d{\bar{\Theta }}^{*}(\zeta ,p)}{d\zeta }\right|}_{\zeta =0^{+}}-Bi\;[{\bar{\Theta }}^{*}(0^{+},\tau )-{\bar{\Theta }}^{*}(0^{-},\tau )]=0,$$

(23) $${\left.\frac{d{\bar{\Theta }}^{*}(\zeta ,p)}{d\zeta }\right|}_{\zeta =1}+Bi_{1}\;{\bar{\Theta }}^{*}(1,p)=0,$$

(24) $${\left.d^{*}\frac{d{\bar{\Theta }}^{*}(\zeta ,p)}{d\zeta }\right|}_{\zeta =-d^{*}}-Bi_{2}\;{\bar{\Theta }}^{*}(-d^{*},p)=0.$$

The general solution of the ordinary differential Equations (19) and (20) is given by the following:

(25) $$\begin{array}{c} {\bar{\Theta }}^{*}(\zeta ,p)=A_{1}(p)\mathrm{sh}(\zeta \sqrt{p})+B_{1}(p)\mathrm{ch}(\zeta \sqrt{p}),\;0\leq \zeta \leq 1, \end{array}$$

(26) $${\bar{\Theta }}^{*}(\zeta ,p)=(\varsigma \sqrt{p})[A_{2}(p)I_{1}(\varsigma \sqrt{p})+B_{2}(p)K_{1}(\varsigma \sqrt{p})],-d^{*}\leq \zeta \leq 0,$$

where

(27) $$\xi =\frac{2\delta }{\gamma^{*}},\;\delta =\frac{d^{*}}{\sqrt{k^{*}}},\;\varsigma =\xi \;e^{\frac{\gamma^{*}\zeta }{2d^{*}}},$$

where $I_{n}(x)$ and $K_{n}(x)$ are the modified Bessel functions of the first and second kinds, respectively, of the n-th order [14], and $A_{l}(p)$ and $B_{l}(p)$, l=1,2, are unknown functions of the Laplace integral transform parameter p (18).

By considering the derivatives [15]

(28) $$\begin{array}{c} sh\prime (x)=\mathrm{ch}(x),\;ch\prime (x)=\mathrm{sh}(x),\;{\;I\prime }_{1}(x)=I_{0}(x)-x^{-1}I_{1}(x),\\ \;K_{1}(x)=-K_{0}(x)-x^{-1}K_{1}(x), \end{array}$$

in solutions (25)–(27), the following was found:

(29) $$\frac{d{\bar{\Theta }}^{*}(\zeta ,p)}{d\zeta }=\sqrt{p}[A_{1}(p)\mathrm{ch}(\zeta \sqrt{p})+B_{1}(p)\mathrm{sh}(\zeta \sqrt{p})],\;0\leq \zeta \leq 1,$$

(30) $$\frac{d{\bar{\Theta }}^{*}(\zeta ,p)}{d\zeta }=\left(\frac{\gamma^{*}\varsigma^{2}p}{2d^{*}}\right)\left[A_{2}(p)I_{0}(\varsigma \sqrt{p})-B_{2}(p)K_{0}(\varsigma \sqrt{p})\right],-d^{*}\leq \zeta \leq 0.$$

Substituting the relations (25), (26) and (29), (30) into the transformed boundary conditions (21)–(24), the following system of four linear equations with respect to the functions $A_{l}(p)$ and $B_{l}(p)$, l=1,2, is obtained:

(31) $$\begin{array}{c} A_{1}(p)-a_{13}(p)A_{2}(p)+a_{14}(p)B_{2}(p)=-{\left(p\sqrt{p}\right)}^{-1},\\ \;A_{1}(p)-a_{22}(p)B_{1}(p)+a_{23}(p)A_{2}(p)-a_{24}(p)B_{2}(p)=0, \end{array}$$

(32) $$a_{31}(p)A_{1}(p)+a_{32}(p)B_{1}(p)=0,\;a_{43}(p)A_{2}(p)-a_{44}(p)B_{2}(p)=0,$$

where

(33) $$a_{13}(p)=\varepsilon (\xi \sqrt{p})I_{0}(\xi \sqrt{p}),\;a_{14}(p)=\varepsilon (\xi \sqrt{p})K_{0}(\xi \sqrt{p}),\;a_{22}(p)=\frac{Bi}{\sqrt{p}},$$

(34) $$\begin{array}{c} a_{23}(p)=(\xi \sqrt{p})\left[\varepsilon \;I_{0}(\xi \sqrt{p})+\frac{Bi}{\sqrt{p}}I_{1}(\xi \sqrt{p})\right],\\ \;a_{24}(p)=(\xi \sqrt{p})\left[\varepsilon \;K_{0}(\xi \sqrt{p})-\frac{Bi}{\sqrt{p}}K_{1}(\xi \sqrt{p})\right], \end{array}$$

(35) $$a_{31}(p)=\mathrm{ch}(\sqrt{p})+\frac{Bi_{1}}{\sqrt{p}}\mathrm{sh}(\sqrt{p}),\;a_{32}(p)=\mathrm{sh}(\sqrt{p})+\frac{Bi_{1}}{\sqrt{p}}\mathrm{ch}(\sqrt{p}),$$

(36) $$a_{43}(p)=\frac{1}{2}\gamma^{*}\eta \;I_{0}(\eta \sqrt{p})-\frac{Bi_{2}}{\sqrt{p}}I_{1}(\eta \sqrt{p}),\;a_{44}(p)=\frac{1}{2}\gamma^{*}\eta \;K_{0}(\eta \sqrt{p})+\frac{Bi_{2}}{\sqrt{p}}K_{1}(\eta \sqrt{p}),$$

(37) $$\eta =\xi \;e^{-0.5\gamma^{*}},\;\varepsilon =\frac{K^{*}}{\sqrt{k^{*}}}.$$

The solution to the system of Equations (31)–(37) has the following form:

(38) $$A_{l}(p)=\;\frac{\Delta_{A_{l}}(p)}{p\sqrt{p}\;\Delta (p)},\;B_{l}(p)=\frac{\Delta_{B_{l}}(p)}{p\sqrt{p}\;\Delta (p)},\;l=1,2,$$

where

(39) $$\begin{array}{c} \Delta_{A_{1}}(p)=-a_{32}(p)\Delta_{4}(p),\;\Delta_{B_{1}}(p)=a_{31}(p)\Delta_{4}(p),\;\Delta_{A_{2}}(p)=a_{44}(p)\Delta_{2}(p),\\ \;\Delta_{B_{2}}(p)=a_{43}(p)\Delta_{2}(p), \end{array}$$

(40) $$\Delta (p)=\Delta_{1}(p)\Delta_{2}(p)+\Delta_{3}(p)\Delta_{4}(p),$$

(41) $$\Delta_{1}(p)=a_{13}(p)a_{44}(p)-a_{14}(p)a_{43}(p),\;\Delta_{2}(p)=a_{22}(p)a_{31}(p)+a_{32}(p),$$

(42) $$\Delta_{3}(p)=a_{32}(p),\;\Delta_{4}(p)=a_{23}(p)a_{44}(p)-a_{24}(p)a_{43}(p).$$

Substituting functions $A_{l}(p)$ and $B_{l}(p)$, l=1,2, from (38)–(42) into solutions (25)–(27), (29) and (30) yields the following:

(43) $${\bar{\Theta }}^{*}(\zeta ,p)=\frac{\Phi_{1}(\zeta ,p)}{p\sqrt{p}\;\;\Delta (p)},\frac{d{\bar{\Theta }}^{*}(\zeta ,p)}{d\zeta }=\frac{Q_{1}(\zeta ,p)}{p\;\;\Delta (p)},\;0\leq \zeta \leq 1,$$

(44) $${\bar{\Theta }}^{*}(\zeta ,p)=\frac{\Phi_{2}(\zeta ,p)}{p\;\Delta (p)},\frac{d{\bar{\Theta }}^{*}(\zeta ,p)}{d\zeta }=\frac{\;Q_{2}(\zeta ,p)}{\sqrt{p}\;\;\Delta (p)},-d^{*}\leq \zeta \leq 0,$$

where

(45) $$\Phi_{1}(\zeta ,p)=\Delta_{A_{1}}(p)\mathrm{sh}(\zeta \sqrt{p})+\Delta_{B_{1}}(p)\mathrm{ch}(\zeta \sqrt{p}),$$

(46) $$\Phi_{2}(\zeta ,p)=\varsigma [\Delta_{A_{2}}(p)I_{1}(\varsigma \sqrt{p})+\Delta_{B_{2}}(p)K_{1}(\varsigma \sqrt{p})],$$

(47) $$Q_{1}(\zeta ,p)=\Delta_{A_{1}}(p)\mathrm{ch}(\zeta \sqrt{p})+\Delta_{B_{1}}(p)\mathrm{sh}(\zeta \sqrt{p}),$$

(48) $$Q_{2}(\zeta ,p)=\left(\frac{\gamma^{*}\varsigma^{2}}{2d^{*}}\right)\left[\Delta_{A_{2}}(p{)I}_{0}(\varsigma \sqrt{p})-\Delta_{B_{2}}(p{)K}_{0}(\varsigma \sqrt{p}\right],$$

The limiting values of the transformed solutions (43)–(48) take the following form:

(49) $${\bar{\Theta }}^{*}(0^{+},p)=\frac{\Delta_{B_{1}}(p)}{p\sqrt{p}\;\Delta (p)},\;{\left.\frac{d{\bar{\Theta }}^{*}(\zeta ,p)}{d\zeta }\right|}_{\zeta =0^{+}}=\frac{\Delta_{A_{1}}(p)}{p\;\Delta (p)},$$

(50) $${\bar{\Theta }}^{*}(0^{-},p)=\frac{\Phi_{2}(0^{-},p)}{p\;\Delta (p)},\;{\left.\frac{d{\bar{\Theta }}^{*}(\zeta ,p)}{d\zeta }\right|}_{\zeta =0^{-}}=\frac{Q_{2}(0^{-},p)}{\sqrt{p}\;\Delta (p)},$$

(51) $${\bar{\Theta }}^{*}(1,p)=\frac{\Phi_{1}(1,p)}{\;p\sqrt{p}\;\Delta (p)},\;{\left.\frac{d{\bar{\Theta }}^{*}(\zeta ,p)}{d\zeta }\right|}_{\zeta =1}=\frac{Q_{1}(1,p)}{p\;\;\Delta (p)},$$

(52) $${\bar{\Theta }}^{*}(-d^{*},p)=\frac{\Phi_{2}(-d^{*},p)}{\;p\;\Delta (p)},\;{\left.\frac{d{\bar{\Theta }}^{*}(\zeta ,p)}{d\zeta }\right|}_{\zeta =-d^{*}}=\frac{\;Q_{2}(-d^{*},p)}{\sqrt{p}\;\;\Delta (p)},$$

where

(53) $$\Phi_{2}(0^{-},p)=\xi [\Delta_{A_{2}}(p)I_{1}(\xi \sqrt{p})+\Delta_{B_{2}}(p)K_{1}(\xi \sqrt{p})],$$

(54) $$Q_{2}(0^{-},p)=\left(\frac{\gamma^{*}\xi^{2}}{2d^{*}}\right)\left[\Delta_{A_{2}}(p{)I}_{0}(\xi \sqrt{p})-\Delta_{B_{2}}(p{)K}_{0}(\xi \sqrt{p})\right],$$

(55) $$\Phi_{1}(1,p)=\Delta_{A_{1}}(p)\mathrm{sh}(\sqrt{p})+\Delta_{B_{1}}(p)\mathrm{ch}(\sqrt{p}),$$

(56) $$Q_{1}(1,p)=\Delta_{A_{1}}(p)\mathrm{ch}(\sqrt{p})+\Delta_{B_{1}}(p)\mathrm{sh}(\sqrt{p}),$$

(57) $$\Phi_{2}(-d^{*},p)=\eta [\Delta_{A_{2}}(p)I_{1}(\eta \sqrt{p})+\Delta_{B_{2}}(p)K_{1}(\eta \sqrt{p})],$$

(58) $$Q_{2}(-d^{*},p)=\left(\frac{\gamma^{*}\eta^{2}}{2d^{*}}\right)\frac{\xi e^{-\gamma^{*}}}{\sqrt{k^{*}}}\left[\Delta_{A_{2}}(p{)I}_{0}(\eta \sqrt{p})-\Delta_{B_{2}}(p{)K}_{0}(\eta \sqrt{p})\right],$$

The parameters ξ and η are determined from (27) and (37), respectively. Incorporating (49)–(58) yields the following:

(59) $$\begin{array}{l} {\left.K^{*}\frac{d{\bar{\Theta }}^{*}(\zeta ,p)}{d\zeta }\right|}_{\zeta =0^{-}}-\;{\left.\frac{d{\bar{\Theta }}^{*}(\zeta ,p)}{d\zeta }\right|}_{\zeta =0^{+}}=\frac{1}{p\Delta (p)}\left[K^{*}\sqrt{p}Q_{2}(0^{-},p)-Q_{1}(0^{+},p)\right]=\\ =\frac{1}{p\Delta (p)}\left\{\varepsilon (\xi \sqrt{p})\left[\Delta_{A_{2}}(p{)I}_{0}(\xi \sqrt{p})-\Delta_{B_{2}}(p{)K}_{0}(\xi \sqrt{p})\right]-\Delta_{A_{1}}(p)\right\}=\\ =\frac{1}{p\Delta (p)}\left\{\left[a_{13}(p)a_{44}(p)-a_{14}(p)a_{43}(p)\right]\Delta_{2}(p)+a_{32}(p)\Delta_{4}(p)\right\}=\\ =\frac{\left[\Delta_{1}(p)\Delta_{2}(p)+\Delta_{3}(p)\Delta_{4}(p)\right]}{p\Delta (p)}=\frac{1}{p}, \end{array}$$

(60) $$\begin{array}{l} {\left.K^{*}\frac{d{\bar{\Theta }}^{*}(\zeta ,p)}{d\zeta }\right|}_{\zeta =0^{-}}+{\left.\frac{d{\bar{\Theta }}^{*}(\zeta ,p)}{d\zeta }\right|}_{\zeta =0^{+}}-Bi\left[{\bar{\Theta }}^{*}(0^{+},\tau )-{\bar{\Theta }}^{*}(0^{-},\tau )\right]\;=\\ =\frac{\Delta_{4}(p)}{p\Delta (p)}\left[-a_{32}(p)-a_{22}(p)a_{31}(p)+a_{22}(p)a_{31}(p)+a_{32}(p)\right]=0, \end{array}$$

(61) $$\begin{array}{l} {\left.\frac{d{\bar{\Theta }}^{*}(\zeta ,p)}{d\zeta }\right|}_{\zeta =1}+Bi_{1}\;{\bar{\Theta }}^{*}(1,p)=\frac{1}{p\Delta (p)}\left[Q_{1}(1,p)+\frac{Bi_{1}}{\sqrt{p}}\Phi_{1}(1,p)\right]=\\ =\frac{1}{p\Delta (p)}\left\{\left[\mathrm{ch}(\sqrt{p})+\frac{Bi_{1}}{\sqrt{p}}\mathrm{sh}(\sqrt{p})\right]\Delta_{A_{1}}(p)+\left[\mathrm{sh}(\sqrt{p})+\frac{Bi_{1}}{\sqrt{p}}\mathrm{ch}(\sqrt{p})\right]\Delta_{B_{1}}(p)\right\}=\\ =\frac{\Delta_{4}(p)}{p\Delta (p)}\left[-a_{31}(p)a_{32}(p)+a_{32}(p)a_{31}(p)\right]=0, \end{array}$$

(62) $$\begin{array}{l} {\left.d^{*}\frac{d{\bar{\Theta }}^{*}(\zeta ,p)}{d\zeta }\right|}_{\zeta =-d^{*}}-Bi_{2}\;{\bar{\Theta }}^{*}(-d^{*},p)=\\ =\frac{\eta \Delta_{2}(p)}{\sqrt{p}\Delta (p)}\left[a_{43}(p)a_{44}(p)-a_{44}(p)a_{43}(p)\right]=0. \end{array}$$

It is verified by relations (59)–(62) that solutions (43)–(48) satisfy boundary conditions (21)–(24) in the Laplace transform domain.

## 4. The Exact Solution in the Time Domain

The dimensionless temperature increments are assumed in the following form:

(63) $$\Theta^{*}(\zeta ,\tau )=\Theta_{1,0}^{*}(\zeta )+\Theta_{1}^{*}(\zeta ,\tau ),\;0\leq \zeta \leq 1,\;\tau \geq 0,$$

(64) $$\Theta^{*}(\zeta ,\tau )=\Theta_{2,0}^{*}(\zeta )+\Theta_{2}^{*}(\zeta ,\tau ),-d^{*}\leq \zeta \leq 0,\;\tau \geq 0.$$

Steady-state components $\Theta_{l,0}^{*}(\zeta )$, l=1, 2, in Formulas (63) and (64) are defined as follows:

(65) $$\Theta_{1,0}^{*}(\zeta )=\underset{p\to 0}{\lim}\frac{\Phi_{1}(\zeta ,p)}{\sqrt{p}\Delta (p)},\;\Theta_{2,0}^{*}(\zeta )=\underset{p\to 0}{\lim}\frac{\Phi_{2}(\zeta ,p)}{\Delta (p)},$$

where functions Δ(p) and $\Phi_{l}(\zeta ,p)$, l=1,2, are described by expressions (40)–(42), (45) and (46), respectively. Considering that for small values of the argument, there is [15]

(66) $$\begin{array}{c} \mathrm{sh}(\sqrt{p})≅\sqrt{p},\;\mathrm{ch}(\sqrt{p})≅1,\;I_{0}(\sqrt{p})≅1,\;I_{1}(\sqrt{p})≅0.5\sqrt{p},\;K_{0}(\sqrt{p})≅-\ln(\sqrt{p}),\\ \;K_{1}(\sqrt{p})≅{\left(\sqrt{p}\right)}^{-1}, \end{array}$$

Equations (33)–(36) yield the following:

(67) $$a_{13}(p)≅\varepsilon (\xi \sqrt{p}),\;a_{14}(p)≅-\varepsilon (\xi \sqrt{p})\ln(\xi \sqrt{p}),$$

(68) $$a_{23}(p)≅(\xi \sqrt{p})\left(\varepsilon \;+\frac{1}{2}\xi Bi\right),\;a_{24}(p)=-a_{22}(p)≅-\frac{Bi}{\sqrt{p}},$$

(69) $$a_{31}(p)≅1+Bi_{1},\;a_{32}(p)≅\sqrt{p}+\frac{Bi_{1}}{\sqrt{p}},$$

(70) $$a_{43}(p)≅\frac{1}{2}\eta (\gamma^{*}-Bi_{2}),\;a_{44}(p)≅-\frac{1}{2}\gamma^{*}\eta \;\ln(\eta \sqrt{p})+\frac{Bi_{2}}{\eta p}.$$

Next, by substituting relations (67)–(70) into Equations (39)–(42), (45), and (46), the following is obtained:

(71) $$\sqrt{p}\Phi_{1}(\zeta ,p)≅\varphi_{1}(\zeta ),\;\;0\leq \zeta \leq 1,\;p\Phi_{2}(\zeta ,p)≅\varphi_{2}(\zeta ),\;\;-d^{*}\leq \zeta \leq 0,\;p\Delta (p)≅\alpha ,$$

where

(72) $$\varphi_{1}(\zeta )=\alpha_{2}[1+Bi_{1}(1-\zeta )],\;\varphi_{2}(\zeta )=\delta \alpha_{1}\{1-Bi_{2}{\left(\gamma^{*}\right)}^{-1}[1-e^{\gamma^{*}\left(1+\frac{\zeta }{d^{*}}\right)}]\},$$

(73) $$\begin{array}{c} \alpha_{1}=Bi_{1}+Bi(1+Bi_{1}),\;\alpha_{2}=\varepsilon Bi_{2}e^{\gamma^{*}}+\delta Bi[1-Bi_{2}{\left(\gamma^{*}\right)}^{-1}(1-e^{\gamma^{*}})],\\ \;\alpha =\alpha_{2}Bi_{1}+\varepsilon \alpha_{1}Bi_{2}e^{\gamma^{*}}. \end{array}$$

Taking into account (72) and (73) in limits (65), the dimensionless steady-state temperature rises take the following form:

(74) $$\Theta_{1,0}^{*}(\zeta )=\alpha^{-1}\varphi_{1}(\zeta ),\;\;0\leq \zeta \leq 1,\;\Theta_{2,0}^{*}(\zeta )=\alpha^{-1}\varphi_{2}(\zeta ),\;\;-d^{*}\leq \zeta \leq 0.$$

To find the steady-state components $\Theta_{l,0}^{*}(\zeta )$, l=1,2, in solutions (63) and (64), the following denotation is introduced:

(75) $$\mu =i\sqrt{p},$$

Then, with the consideration of the following relations [14,15],

(76) $$\mathrm{sh}(x)=-i\sin(ix),\;\mathrm{ch}(x)=\cos(ix),\;I_{0}(x)=J_{0}(ix),\;I_{1}(x)=-iJ_{1}(ix),\;i\equiv \sqrt{-1},$$

(77) $$K_{0}(x)=-0.5\pi [Y_{0}(ix)-iJ_{0}(ix)],\;K_{1}(x)=-0.5\pi [J_{1}(ix)+iY_{1}(ix)]$$

where $J_{n}(x)$ and $Y_{n}(x)$, n=0,1, are Bessel functions of the first and second kinds of the *n*-th order, from Formulas (39)–(42), (45) and (46), the following is obtained:

(78) $$\Phi_{1}(\zeta ,p)=i0.5\pi (\xi \mu )\Phi_{1}^{*}(\zeta ,\mu ),\;\Phi_{2}(\zeta ,p)=i0.5\pi (\xi \mu )e^{\frac{\gamma^{*}\zeta }{2d^{*}}}\Phi_{2}^{*}(\zeta ,\mu ),\;\Delta (p)=0.5\pi (\xi \mu )\Psi^{*}(\mu ),$$

where

(79) $$\Phi_{1}^{*}(\zeta ,\mu )=\chi_{1}(\zeta ,\mu )c_{2}(\mu ),\;\Phi_{2}^{*}(\zeta ,\mu )=\chi_{2}(\zeta ,\mu )c_{1}(\mu ),$$

(80) $$\Psi^{*}(\mu )=a_{1}(\mu )c_{2}(\mu )+\varepsilon a_{2}(\mu )c_{1}(\mu ),$$

(81) $$\begin{array}{c} \chi_{1}(\zeta ,\mu )=a_{1}(\mu )\sin(\zeta \mu )+b_{1}(\mu )\cos(\zeta \mu ),\\ \;\chi_{2}(\zeta ,\mu )=\Phi_{0,1}(\eta \mu ,\varsigma \mu )-Bi_{2}^{*}\mu^{-1}\Phi_{1,1}(\eta \mu ,\varsigma \mu ), \end{array}$$

(82) $$a_{1}(\mu )=\sin\mu -Bi_{1}\mu^{-1}\cos\mu ,\;b_{1}(\mu )=\cos\mu +Bi_{1}\mu^{-1}\sin\mu ,$$

(83) $$\begin{array}{c} a_{2}(\mu )=\Phi_{0,0}(\xi \mu ,\eta \mu )-Bi_{2}^{*}\mu^{-1}\Phi_{0,1}(\xi \mu ,\eta \mu ),\\ \;b_{2}(\mu )=\Phi_{1,0}(\xi \mu ,\eta \mu )-Bi_{2}^{*}\mu^{-1}\Phi_{1,1}(\xi \mu ,\eta \mu ), \end{array}$$

(84) $$c_{1}(\mu )=a_{1}(\mu )-Bi\;\mu^{-1}b_{1}(\mu ),\;c_{2}(\mu )=\varepsilon a_{2}(\mu )+Bi\;\mu^{-1}b_{2}(\mu ),$$

(85) $$Bi_{2}^{*}=\frac{2}{\gamma^{*}\eta }Bi_{2},$$

where parameters ξ, η, ς and ε are determined by Expressions (27) and (37), and the function $\Phi_{m,n}(x,y)$, m,n=0,1, is defined as follows:

(86) $$\Phi_{0,0}(x,y)=J_{0}(x)Y_{0}(y)-Y_{0}(x)J_{0}(y),\;\Phi_{0,1}(x,y)=J_{0}(x)Y_{1}(y)-Y_{0}(x)J_{1}(y),$$

(87) $$\Phi_{1,0}(x,y)=J_{1}(x)Y_{0}(y)-Y_{1}(x)J_{0}(y),\;\Phi_{1,1}(x,y)=J_{1}(x)Y_{1}(y)-Y_{1}(x)J_{1}(y).$$

By incorporating Equations (78)–(87) and applying the expansion theorem [16], the following is obtained

(88) $$\Theta_{1}^{*}(\zeta ,\tau )=-2\sum_{n=1}^{\infty }\frac{\Phi_{1}^{*}(\zeta ,\mu_{n})}{\mu_{n}^{2}\;\Psi^{{*}^{\prime }}(\mu_{n})}e^{-\mu_{n}^{2}\tau },\;\Theta_{2}^{*}(\zeta ,\tau )=-2e^{\frac{\gamma^{*}\zeta }{2d^{*}}}\sum_{n=1}^{\infty }\frac{\Phi_{2}^{*}(\zeta ,\mu_{n})}{\mu_{n}^{2}\;{\tilde{\Psi }}^{*}(\mu_{n})}e^{-\mu_{n}^{2}\tau },$$

where $\mu_{n}$, n=1,2,…, are the simple positive roots of the following characteristic equation:

(89) $$\Psi^{*}(\mu )=0,$$

with function $\Psi^{*}(\mu )$ given by (81). The derivative of this function, contained in the denominator of (88), has the following form:

(90) $${\tilde{\Psi }}^{*}(\mu )\equiv d\Psi^{*}(\mu )/d\mu ={\tilde{a}}_{1}(\mu )c_{2}(\mu )+a_{1}(\mu ){\tilde{c}}_{2}(\mu )+\varepsilon [{\tilde{a}}_{2}(\mu )c_{1}(\mu )+a_{2}(\mu ){\tilde{c}}_{1}(\mu )],$$

where by using Equations (82)–(87), the following are found:

(91) $${\tilde{a}}_{1}(\mu )=b_{1}(\mu )+Bi_{1}\mu^{-2}\cos\mu ,\;{\tilde{b}}_{1}(\mu )=-a_{1}(\mu )-Bi_{1}\mu^{-2}\sin\mu ,$$

(92) $${\tilde{a}}_{2}(\mu )={\tilde{\Phi }}_{0,0}(\xi \mu ,\eta \mu )-Bi_{2}^{*}\mu^{-1}[{\tilde{\Phi }}_{0,1}(\xi \mu ,\eta \mu )-\mu^{-1}\Phi_{0,1}(\xi \mu ,\eta \mu )],$$

(93) $${\tilde{b}}_{2}(\mu )={\tilde{\Phi }}_{1,0}(\xi \mu ,\eta \mu )-Bi_{2}^{*}\mu^{-1}[{\tilde{\Phi }}_{1,1}(\xi \mu ,\eta \mu )-\mu^{-1}\Phi_{1,1}(\xi \mu ,\eta \mu )],$$

(94) $${\tilde{c}}_{1}(\mu )={\tilde{a}}_{1}(\mu )-Bi\mu^{-1}[{\tilde{b}}_{1}(\mu )-\mu^{-1}b_{1}(\mu )],\;{\tilde{c}}_{2}(\mu )=\varepsilon {\tilde{a}}_{2}(\mu )+Bi\mu^{-1}[{\tilde{b}}_{2}(\mu )-\mu^{-1}b_{2}(\mu )],$$

(95) $${\tilde{\Phi }}_{0,0}(\xi \mu ,\eta \mu )=-\xi \Phi_{1,0}(\xi \mu ,\eta \mu )-\eta \Phi_{0,1}(\xi \mu ,\eta \mu ),$$

(96) $${\tilde{\Phi }}_{0,1}(\xi \mu ,\eta \mu )=-\xi \Phi_{1,1}(\xi \mu ,\eta \mu )+\eta \Phi_{0,0}(\xi \mu ,\eta \mu )-\mu^{-1}\Phi_{0,1}(\xi \mu ,\eta \mu ),$$

(97) $${\tilde{\Phi }}_{1,0}(\xi \mu ,\eta \mu )=\xi \Phi_{0,0}(\xi \mu ,\eta \mu )-\eta \Phi_{1,1}(\xi \mu ,\eta \mu )-\mu^{-1}\Phi_{1,0}(\xi \mu ,\eta \mu ),$$

(98) $${\tilde{\Phi }}_{1,1}(\xi \mu ,\eta \mu )=\xi \Phi_{0,1}(\xi \mu ,\eta \mu )+\eta \Phi_{1,0}(\xi \mu ,\eta \mu )-2\mu^{-1}\Phi_{1,1}(\xi \mu ,\eta \mu ).$$

Thus, the analytical solution to the thermal friction problem (11)–(17) is given by (63) and (64), consisting of the steady-state $\Theta_{l,0}^{*}(\zeta )$ (74) and transient $\Theta_{l}^{*}(\zeta ,\tau )$, l=1, 2 (88) components.

Based on Fourier’s law [17], the dimensionless intensities of the heat fluxes, generated at the contact surface and directed along the normal to the layers’ interior, are defined as follows:

(99) $$q^{*}(\zeta ,\tau )=-\frac{\partial \Theta^{*}(\zeta ,\tau )}{\partial \zeta },\;0\leq \zeta \leq 1,\;\;\tau \geq 0,\;q^{*}(\zeta ,\tau )=K^{*}\frac{\partial \Theta^{*}(\zeta ,\tau )}{\partial \zeta },\;-d^{*}\leq \zeta \leq 0,\;\tau \geq 0.$$

By considering the form of solutions (63) and (64), Equation (99) is presented as follows:

(100) $$\begin{array}{c} q^{*}(\zeta ,\tau )=q_{1,0}^{*}(\zeta )+q_{1}^{*}(\zeta ,\tau ),\;\;0\leq \zeta \leq 1,\;\;\tau \geq 0,\\ \;q^{*}(\zeta ,\tau )=q_{2,0}^{*}(\zeta )+q_{2}^{*}(\zeta ,\tau ),\;\;-d^{*}\leq \zeta \leq 0,\;\;\tau \geq 0, \end{array}$$

where the steady-state components are determined after differentiating solutions (72)–(74) in the following form:

(101) $$q_{1,0}^{*}(\zeta )\equiv -\alpha^{-1}{\varphi^{\prime }}_{1}(\zeta )=\alpha^{-1}\alpha_{2}Bi_{1},\;q_{2,0}^{*}(\zeta )=K^{*}\alpha^{-1}{\varphi^{\prime }}_{2}(\zeta )=\alpha^{-1}\varepsilon \alpha_{1}Bi_{2}e^{\gamma^{*}\left(1+\frac{\zeta }{d^{*}}\right)}.$$

Taking into account the derivatives of functions $\Phi_{l}^{*}(\zeta ,\mu_{n}),\;\;l=1,2$ (79)–(85) with respect to the spatial coordinate ζ in solution (88),

(102) $$\Phi_{1}^{*\prime }(\zeta ,\mu_{n})=c_{2}(\mu_{n}){\chi^{\prime }}_{1}(\zeta ,\mu_{n}),\;\Phi_{2}^{*\prime }(\zeta ,\mu_{n})=c_{1}(\mu_{n}){\chi^{\prime }}_{2}(\zeta ,\mu_{n}),$$

(103) $$\begin{array}{c} {\chi^{\prime }}_{1}(\zeta ,\mu_{n})=\mu_{n}[a_{1}(\mu_{n})\cos(\zeta \mu_{n})-b_{1}(\mu_{n})\sin(\zeta \mu_{n})],\\ \;{\chi^{\prime }}_{2}(\zeta ,\mu_{n})={\Phi^{\prime }}_{0,1}(\eta \mu_{n},\varsigma \mu_{n})-Bi_{2}^{*}\mu_{n}^{-1}{\Phi^{\prime }}_{1,1}(\eta \mu_{n},\varsigma \mu_{n}), \end{array}$$

(104) $${\Phi^{\prime }}_{0,1}(\eta \mu_{n},\varsigma \mu_{n})={\left(\xi \sqrt{k^{*}}\right)}^{-1}[\varsigma \mu_{n}\Phi_{0,0}(\eta \mu_{n},\varsigma \mu_{n})-\Phi_{0,1}(\eta \mu_{n},\varsigma \mu_{n})].$$

(105) $${\Phi^{\prime }}_{1,1}(\eta \mu_{n},\varsigma \mu_{n})={\left(\xi \sqrt{k^{*}}\right)}^{-1}[\varsigma \mu_{n}\Phi_{1,0}(\eta \mu_{n},\varsigma \mu_{n})-\Phi_{1,1}(\eta \mu_{n},\varsigma \mu_{n})].$$

the transient components of the dimensionless intensities of the heat fluxes (100) are determined as follows:

(106) $$q_{1}^{*}(\zeta ,\tau )=2\sum_{n=1}^{\infty }\frac{Q_{1}^{*}(\zeta ,\mu_{n})}{\mu_{n}\;{\tilde{\Psi }}^{*}(\mu_{n})}e^{-\mu_{n}^{2}\tau },\;0\leq \zeta \leq 1,\;\;\tau \geq 0,$$

(107) $$q_{2}^{*}(\zeta ,\tau )=-2\varepsilon e^{\frac{\gamma^{*}\zeta }{d^{*}}}\sum_{n=1}^{\infty }\frac{Q_{2}^{*}(\zeta ,\mu_{n})}{\mu_{n}\;{\tilde{\Psi }}^{*}(\mu_{n})}e^{-\mu_{n}^{2}\tau },\;\;-d^{*}\leq \zeta \leq 0,\;\;\tau \geq 0.$$

where

(108) $$Q_{1}^{*}(\zeta ,\mu_{n})=[a_{1}(\mu_{n})\cos(\zeta \mu_{n})-b_{1}(\mu_{n})\sin(\zeta \mu_{n})]c_{2}(\mu_{n}).$$

(109) $$Q_{2}^{*}(\zeta ,\mu_{n})=[\Phi_{0,0}(\eta \mu_{n},\varsigma \mu_{n})-Bi_{2}^{*}\mu_{n}^{-1}\Phi_{1,0}(\eta \mu_{n},\varsigma \mu_{n})]c_{1}(\mu_{n}).$$

## 5. Temperature and Heat Flux Intensities at the Layer Surfaces

Friction surfaces. The maximum temperature is reached on the friction surfaces of the layers $\zeta =0^{\pm }$. It follows from Equations (72) and (74) that the steady-state components of the dimensionless temperature rise on these surfaces are equal to the following:

(110) $$\Theta_{1,0}^{*}(0^{+})=\alpha^{-1}\alpha_{2}(1+Bi_{1}),\;\Theta_{2,0}^{*}(0^{-})=\alpha^{-1}\delta \alpha_{1}[1-Bi_{2}{\left(\gamma^{*}\right)}^{-1}(1-e^{\gamma^{*}})],$$

and coefficients $\alpha_{l},\;l=1,\;2$ and α are calculated from expression (73).

Next, by substituting $\zeta =0^{\pm }$ into Equation (88), the transient components of the maximum temperature rise are written in the following form:

(111) $$\Theta_{1,0}^{*}(0^{+})=\alpha^{-1}\alpha_{2}(1+Bi_{1}),\;\Theta_{2,0}^{*}(0^{-})=\alpha^{-1}\delta \alpha_{1}[1-Bi_{2}{\left(\gamma^{*}\right)}^{-1}(1-e^{\gamma^{*}})],$$

where

(112) $$\Phi_{1}^{*}(0^{+},\mu_{n})=b_{1}(\mu_{n})c_{2}(\mu_{n}),\;\Phi_{2}^{*}(0^{-},\mu_{n})=-b_{2}(\mu_{n})c_{1}(\mu_{n}).$$

By substituting $\zeta =0^{\pm }$ into Equations (101) and (106)–(109), the following is obtained:

(113) $$q_{1,0}^{*}(0^{+})=\alpha^{-1}\alpha_{2}Bi_{1},\;q_{2,0}^{*}(0^{-})=\alpha^{-1}\varepsilon \alpha_{1}Bi_{2}e^{\gamma^{*}},$$

(114) $$q_{1}^{*}(0^{+},\tau )=2\sum_{n=1}^{\infty }\frac{Q_{1}^{*}(0^{+},\mu_{n})}{\mu_{n}\;{\tilde{\Psi }}^{*}(\mu_{n})}e^{-\mu_{n}^{2}\tau },\;q_{2}^{*}(0^{-},\tau )=-2\varepsilon \sum_{n=1}^{\infty }\frac{Q_{2}^{*}(0^{-},\mu_{n})}{\mu_{n}\;{\tilde{\Psi }}^{*}(\mu_{n})}e^{-\mu_{n}^{2}\tau },\;\tau \geq 0,$$

where

(115) $$Q_{1}^{*}(0^{+},\mu_{n})=a_{1}(\mu_{n})c_{2}(\mu_{n}),\;Q_{2}^{*}(0^{-},\mu_{n})=-a_{2}(\mu_{n})c_{1}(\mu_{n}).$$

Thus, the dimensionless maximum temperature rises and heat flux intensities on the friction surfaces of the layers are determined from the following formulas

(116) $$\Theta^{*}(0^{+},\tau )=\Theta_{1,0}^{*}(0^{+})+\Theta_{1}^{*}(0^{+},\tau ),\;q^{*}(0^{+},\tau )=q_{1,0}^{*}(0^{+})+q_{1}^{*}(0^{+},\tau ),\;\;\tau \geq 0,$$

(117) $$\Theta^{*}(0^{-},\tau )=\Theta_{2,0}^{*}(0^{-})+\Theta_{2}^{*}(0^{-},\tau ),\;q^{*}(0^{-},\tau )=q_{2,0}^{*}(0^{-})+q_{2}^{*}(0^{-},\tau ),\;\;\tau \geq 0,$$

in which the corresponding components are given by (110)–(115).

Free surfaces. The steady-state components of the dimensionless temperature rises and heat flux intensities on the free surfaces of the upper layer ζ=1 and the lower layer $\zeta =-d^{*}$ are found from (72)–(74) and (101) in the following form:

(118) $$\Theta_{1,0}^{*}(1)=\alpha^{-1}\alpha_{2},\;q_{1,0}^{*}(1)=\alpha^{-1}\alpha_{2}Bi_{1},$$

(119) $$\Theta_{2,0}^{*}(-d^{*})=\alpha^{-1}\delta \alpha_{1},\;q_{2,0}^{*}(-d^{*})=\alpha^{-1}\varepsilon \alpha_{1}Bi_{2}.$$

On the other hand, from Equations (88), (106) and (107), the corresponding transient components are found:

(120) $$\Theta_{1}^{*}(1,\tau )=-2\sum_{n=1}^{\infty }\frac{\Phi_{1}^{*}(1,\mu_{n})}{\mu_{n}^{2}\;{\tilde{\Psi }}^{*}(\mu_{n})}e^{-\mu_{n}^{2}\tau },\;q_{1}^{*}(1,\tau )=2\sum_{n=1}^{\infty }\frac{Q_{1}^{*}(1,\mu_{n})}{\mu_{n}\;{\tilde{\Psi }}^{*}(\mu_{n})}e^{-\mu_{n}^{2}\tau },\;\tau \geq 0,$$

(121) $$\Theta_{2}^{*}(-d^{*},\tau )=-2e^{-0.5\gamma^{*}}\sum_{n=1}^{\infty }\frac{\Phi_{2}^{*}(-d^{*},\mu_{n})}{\mu_{n}^{2}\;{\tilde{\Psi }}^{*}(\mu_{n})}e^{-\mu_{n}^{2}\tau },\;q_{2}^{*}(-d^{*},\tau )=-2\varepsilon e^{-\gamma^{*}}\sum_{n=1}^{\infty }\frac{Q_{2}^{*}(-d^{*},\mu_{n})}{\mu_{n}\;{\tilde{\Psi }}^{*}(\mu_{n})}e^{-\mu_{n}^{2}\tau },\;\tau \geq 0,$$

where

(122) $$\begin{array}{c} \Phi_{1}^{*}(1,\mu_{n})=[a_{1}(\mu_{n})\sin\mu_{n}+b_{1}(\mu_{n})\cos\mu_{n}]c_{2}(\mu_{n}),\\ \;Q_{1}^{*}(1,\mu_{n})=[a_{1}(\mu_{n})\cos\mu_{n}-b_{1}(\mu_{n})\sin\mu_{n}]c_{2}(\mu_{n}), \end{array}$$

(123) $$\Phi_{2}^{*}(-d^{*},\mu_{n})=\Phi_{0,1}(\eta \mu_{n},\eta \mu_{n})c_{1}(\mu_{n}),\;Q_{2}^{*}(-d^{*},\mu_{n})=Bi_{2}^{*}\mu_{n}^{-1}\Phi_{2}^{*}(-d^{*},\mu_{n}).$$

Thus, the dimensionless temperature rises and heat flux intensities on the free surfaces ζ=1 and $\zeta =-d^{*}$ are determined as follows:

(124) $$\Theta^{*}(1,\tau )=\Theta_{1,0}^{*}(1)+\Theta_{1}^{*}(1,\tau ),\;q^{*}(1,\tau )=q_{1,0}^{*}(1)+q_{1}^{*}(1,\tau ),\;\;\tau \geq 0,$$

(125) $$\Theta^{*}(-d^{*},\tau )=\Theta_{2,0}^{*}(-d^{*})+\Theta_{2}^{*}(-d^{*},\tau ),\;q^{*}(-d^{*},\tau )=q_{2,0}^{*}(-d^{*})+q_{2}^{*}(-d^{*},\tau ),\;\;\tau \geq 0,$$

where the corresponding components are given by (118)–(123).

Verification of the boundary conditions. The solutions presented above for the friction and free surfaces of the layers allow for the verification of the boundary conditions (13)–(16). Due to the form of these solutions (116), (117), (124) and (125), this verification is carried out separately for the steady-state and transient components. By substituting the steady-state components (110), (113), (118) and (119) into the boundary conditions (13)–(16), the following identities are obtained:

(126) $$q_{2,0}^{*}(0^{-})+q_{1,0}^{*}(0^{+})=\alpha^{-1}(\varepsilon \alpha_{1}Bi_{2}e^{\gamma^{*}}+\alpha_{2}Bi_{1})\equiv 1,$$

(127) $$\begin{array}{l} q_{2,0}^{*}(0^{-})-q_{1,0}^{*}(0^{+})-Bi[\Theta_{1,0}^{*}(0^{+})-\Theta_{2,0}^{*}(0^{-})]=\\ =\alpha^{-1}\{\varepsilon \alpha_{1}Bi_{2}e^{\gamma^{*}}-\alpha_{2}Bi_{1}-\alpha_{2}Bi(1+Bi_{1})+\delta \alpha_{1}Bi[1-Bi_{2}{\left(\gamma^{*}\right)}^{-1}(1-e^{\gamma^{*}})]\}\equiv 0, \end{array}$$

(128) $$q_{1,0}^{*}(1)-Bi_{1}\Theta_{1,0}^{*}(1)=\alpha^{-1}(\alpha_{2}Bi_{1}-Bi_{1}\alpha_{2})\equiv 0,$$

(129) $${\left(K^{*}\right)}^{-1}d^{*}q_{2,0}^{*}(-d^{*})-Bi_{2}\Theta_{2,0}^{*}(-d^{*})=\alpha^{-1}\delta (\alpha_{1}Bi_{2}-Bi_{2}\alpha_{1})\equiv 0,$$

Taking into account the forms of the dimensionless temperature rise and heat flux intensities (111), (112), (114), (115) and (120)–(123), their fulfillment of the boundary conditions (13)–(16) is equivalent to establishing the following identities:

(130) $$Q_{1}^{*}(0^{+},\mu_{n})-\varepsilon Q_{2}^{*}(0^{-},\mu_{n})=a_{1}(\mu_{n})c_{2}(\mu_{n})+\varepsilon a_{2}(\mu_{n})c_{1}(\mu_{n})=\Psi^{*}(\mu_{n})\equiv 0,$$

(131) $$\begin{array}{c} \mu_{n}Q_{1}^{*}(1,\mu_{n})+Bi_{1}\Phi_{1}^{*}(1,\mu_{n})=\\ \{[a_{1}(\mu_{n})\cos\mu_{n}-b_{1}(\mu_{n})\sin\mu_{n}]+Bi_{1}\mu_{n}^{-1}[a_{1}(\mu_{n})\sin\mu_{n}+b_{1}(\mu_{n})\cos\mu_{n}]\}\mu_{n}c_{2}(\mu_{n})=\\ \;\;\;\;\;\;\;\;\;\;\;\;\;\;\;\;\;\;\;\;\;\;\;\;\;\;\;\;\;\;\;\;\;\;\;\;\;=[a_{1}(\mu_{n})b_{1}(\mu_{n})-b_{1}(\mu_{n})a_{1}(\mu_{n})]\mu_{n}c_{2}(\mu_{n})\equiv 0\;, \end{array}$$

(132) $$\begin{array}{c} \mu_{n}Q_{1}^{*}(1,\mu_{n})+Bi_{1}\Phi_{1}^{*}(1,\mu_{n})=\\ \{[a_{1}(\mu_{n})\cos\mu_{n}-b_{1}(\mu_{n})\sin\mu_{n}]+Bi_{1}\mu_{n}^{-1,}[a_{1}(\mu_{n})\sin\mu_{n}+b_{1}(\mu_{n})\cos\mu_{n}]\}\mu_{n}c_{2}(\mu_{n})=\\ \;\;\;\;\;\;\;\;\;\;\;\;\;\;\;\;\;\;\;\;\;\;\;\;\;\;\;\;\;\;\;\;\;\;\;\;\;=[a_{1}(\mu_{n})b_{1}(\mu_{n})-b_{1}(\mu_{n})a_{1}(\mu_{n})]\mu_{n}c_{2}(\mu_{n})\equiv 0\;, \end{array}$$

(133) $$\mu_{n}\delta e^{-\gamma^{*}}Q_{2}^{*}(-d^{*},\mu_{n})-Bi_{2}e^{-0.5\gamma^{*}}\Phi_{2}^{*}(-d^{*},\mu_{n})=(\delta e^{-0.5\gamma^{*}}Bi_{2}^{*}-Bi_{2})e^{-0.5\gamma^{*}}\Phi_{2}^{*}(-d^{*},\mu_{n})\equiv 0.$$

Identities (126)–(133) demonstrate that solutions (116), (117) and (124), (125) satisfy the boundary conditions (13)–(16). Compliance with the initial condition (17) is carried out numerically by verifying the following equality:

(134) $$\Theta_{l,0}^{*}(\zeta )+\Theta_{l}^{*}(\zeta ,0)=0,\;-d^{*}\leq \zeta \leq 1,\;l=1,\;2.$$

## 6. Some Special Cases of the Solutions

Perfect (full) thermal contact occurs when the sliding surfaces are sufficiently smooth for the contact thermal resistance to be ignored. In this mathematical model, such a case is represented by an infinite thermal contact conductance h→∞ (Bi→∞). Consequently, according to the boundary condition (4) or (14), the temperature rises on the friction surfaces must be identical. By applying the limit Bi→∞ to Equation (73), the solutions (110) yield the following:

(135) $$\Theta_{0}^{*}\equiv \Theta_{1,0}^{*}=\Theta_{2,0}^{*}=\alpha^{-1}\alpha_{1}\alpha_{2},\;\alpha =\alpha_{2}Bi_{1}+\varepsilon \alpha_{1}Bi_{2}e^{\gamma^{*}},\;\alpha_{1}=1+Bi_{1},\;\alpha_{2}=\delta [1-Bi_{2}{\left(\gamma^{*}\right)}^{-1}(1-e^{\gamma^{*}})].$$

In a similar manner, solutions (111) and (112) for Bi→∞ are written in the following form:

(136) $$\Theta^{*}(\tau )\equiv \Theta_{1}^{*}(\tau )=\Theta_{2}^{*}(\tau )=-2\sum_{n=1}^{\infty }\frac{\Phi^{*}(\mu_{n})}{\mu_{n}^{2}\;{\tilde{\Psi }}^{*}(\mu_{n})}e^{-\mu_{n}^{2}\tau },\;\tau \geq 0,$$

where

(137) $$\Phi^{*}(\mu )\equiv \Phi_{1}^{*}(0^{+},\mu )=\Phi_{2}^{*}(0^{-},\mu )=b_{1}(\mu )b_{2}(\mu ),$$

(138) $${\tilde{\Psi }}^{*}(\mu )={\tilde{a}}_{1}(\mu )b_{2}(\mu )+a_{1}(\mu )[{\tilde{b}}_{2}(\mu )-\mu^{-1}b_{2}(\mu )]-\varepsilon \{{\tilde{a}}_{2}(\mu )b_{1}(\mu )+a_{2}(\mu )[{\tilde{b}}_{1}(\mu )-\mu^{-1}b_{1}(\mu )]\},$$

where $\mu_{n}>0$, and n=1,2,3,…, are the roots of the equation:

(139) $$\Psi^{*}(\mu )=a_{1}(\mu )b_{2}(\mu )-\varepsilon a_{2}(\mu )b_{1}(\mu )=0.$$

The functions $a_{l}(\mu )$ and $b_{l}(\mu )$ and their derivatives ${\tilde{a}}_{l}(\mu )$ and ${\tilde{b}}_{l}(\mu )$, l=1,2, are determined from (82), (83) and (91)–(93). Thus, the dimensionless maximum temperature rise under the perfect thermal contact of friction is calculated in the following form:

(140) $$\Theta_{\max}^{*}(\tau )\equiv \Theta_{0}^{*}+\Theta^{*}(\tau ),\;\tau \geq 0,$$

with the components determined from (135)–(139).

Asymptotic solution for small values of the Fourier number. To this end, an analysis of solutions (43)–(48) is carried out for large values of the Laplace transform parameter *p* (18). After considering the behavior of hyperbolic functions and modified Bessel functions for large values of the argument [15],

(141) $$\mathrm{sh}(x)=\mathrm{ch}(x)≅\frac{1}{2}e^{x},\;I_{n}(x)≅\frac{e^{x}}{\sqrt{2\pi x}},\;K_{n}(x)≅\sqrt{\frac{\pi }{2x}\;}e^{-x},\;n=0,1,2,\ldots ,$$

and in Equations (33)–(42), as well as (45) and (46), the following were found:

(142) $$\Phi_{1}(\zeta ,p)≅=a_{1}(p)a_{2}(p)\left(\varepsilon +\frac{Bi}{\sqrt{p}}\right)e^{(1+\xi -\eta -\zeta )\sqrt{p}},\;0\leq \zeta \leq 1,$$

(143) $$\Phi_{2}(\zeta ,p)≅=a_{1}(p)a_{2}(p)\left(1+\frac{Bi}{\sqrt{p}}\right)e^{(1+\varsigma -\eta )\sqrt{p}},-d^{*}\leq \zeta \leq 0,$$

(144) $$\Delta (p)≅a_{1}(p)a_{2}(p)\left[2\varepsilon +(1+\varepsilon )\frac{Bi}{\sqrt{p}}\right]e^{(1+\xi -\eta )\sqrt{p}},$$

(145) $$a_{1}(p)≅\frac{1}{2}\left(1+\frac{Bi_{1}}{\sqrt{p}}\right),\;a_{2}(p)≅\frac{1}{2}e^{0.25\gamma^{*}}\left(\delta \;e^{-0.5\gamma^{*}}+\frac{Bi_{2}}{\sqrt{p}}\right),$$

where parameters ξ, δ, ς and η are defined by (27) and (37). Taking into account the asymptotes (142)–(145), the transformed solutions (43) and (44) are written in the following form:

(146) $${\bar{\Theta }}^{*}(\zeta ,p)≅\frac{1}{2\varepsilon }\left[\frac{\;\varepsilon }{p(\sqrt{p}+\kappa )}+\frac{Bi\;}{p\sqrt{p}(\sqrt{p}+\kappa )}\right]e^{-\zeta \sqrt{p}},\;\;0\leq \zeta \leq 1,$$

(147) $${\bar{\Theta }}^{*}(\zeta ,p)≅\frac{e^{\frac{\gamma^{*}\zeta }{4d^{*}}}}{2\varepsilon }\left[\frac{\;1}{p(\sqrt{p}+\kappa )}+\frac{Bi\;}{p\sqrt{p}(\sqrt{p}+\kappa )}\right]e^{-\zeta_{2}\sqrt{p}},\;\;-d^{*}\leq \zeta \leq 0,$$

where

(148) $$\kappa =\frac{\left(1+\varepsilon \right)}{2\varepsilon }Bi,\;\zeta_{2}=\xi -\varsigma =\frac{2d^{*}}{\gamma^{*}\sqrt{k^{*}}}(1-e^{\frac{\gamma^{*}\zeta }{2d^{*}}})\geq 0.$$

Using the relations [18]

(149) $$L^{-1}\left[\frac{\kappa \;e^{-a\sqrt{p}}}{p(\sqrt{p}+\kappa )};\;\tau \right]=\mathrm{erfc}\left(\frac{a}{2\sqrt{\tau }}\right)-e^{\kappa^{2}\tau +\kappa a}\mathrm{erfc}\left(\frac{a}{2\sqrt{\tau }}+\kappa \sqrt{\tau }\right),\;a\geq 0,$$

(150) $$L^{-1}\left[\frac{\kappa \;e^{-a\sqrt{p}}}{p\sqrt{p}(\sqrt{p}+\kappa )};\;\tau \right]=2\sqrt{\tau }\;\mathrm{ierfc}\;\left(\frac{a}{2\sqrt{\tau }}\right)-\frac{1}{\kappa }\left[\mathrm{erfc}\left(\frac{a}{2\sqrt{\tau }}\right)-e^{\kappa^{2}\tau +\kappa a}\mathrm{erfc}\left(\frac{a}{2\sqrt{\tau }}+\kappa \sqrt{\tau }\right)\right],$$

where $\mathrm{ierfc}(x)=\pi^{-\frac{1}{2}}e^{-x^{2}}-x\;\mathrm{erfc}(x)$ and erfc(x)=1−erf(x), erf(x) are Gauss error functions [15], from Equations (146) and (147), the following asymptotic solutions are obtained to estimate the dimensionless temperature rises in the layers at the initial moments of heating (0≤τ<<1):

(151) $$\Theta^{*}(\zeta ,\tau )≅\frac{2\sqrt{\tau }}{\left(1+\varepsilon \right)}\;\mathrm{ierfc}\;\left(\frac{\zeta }{2\sqrt{\tau }}\right)-\frac{\lambda }{2\kappa }\left[\mathrm{erfc}\left(\frac{\zeta }{2\sqrt{\tau }}\right)-e^{\kappa^{2}\tau +\kappa \zeta }\mathrm{erfc}\left(\frac{\zeta }{2\sqrt{\tau }}+\kappa \sqrt{\tau }\right)\right],\;\;0\leq \zeta \leq 1,$$

(152) $$\begin{array}{c} \Theta^{*}(\zeta ,\tau )≅e^{\frac{\gamma^{*}\zeta }{4d^{*}}}\left\{\frac{2\sqrt{\tau }}{\left(1+\varepsilon \right)}\;\mathrm{ierfc}\;\left(\frac{\zeta_{2}}{2\sqrt{\tau }}\right)+\frac{\lambda }{2\varepsilon \kappa }\left[\mathrm{erfc}\;\left(\frac{\zeta_{2}}{2\sqrt{\tau }}\right)-e^{\kappa^{2}\tau +\kappa \zeta_{2}}\mathrm{erfc}\left(\frac{\zeta_{2}}{2\sqrt{\tau }}+\kappa \sqrt{\tau }\right)\right]\right\},\\ -d^{*}\leq \zeta \leq 0, \end{array}$$

where

(153) $$\lambda =\frac{1-\varepsilon }{1+\varepsilon }.$$

In the case of a homogeneous material of the lower layer ($\gamma^{*}\to 0$), solution (152) takes the following known form [4]:

(154) $$\Theta^{*}(\zeta ,\tau )≅\frac{2\sqrt{\tau }}{\left(1+\varepsilon \right)}\;\mathrm{ierfc}\;\left(\frac{\zeta_{2}}{2\sqrt{\tau }}\right)+\frac{\lambda }{2\varepsilon \kappa }\left[\mathrm{erfc}\;\left(\frac{\zeta_{2}}{2\sqrt{\tau }}\right)-e^{\kappa^{2}\tau +\kappa \zeta_{2}}\mathrm{erfc}\left(\frac{\zeta_{2}}{2\sqrt{\tau }}+\kappa \sqrt{\tau }\right)\right],\;\zeta_{2}=-\frac{\zeta }{\sqrt{k^{*}}},\;\;-d^{*}\leq \zeta \leq 0.$$

It should be noted that solutions (151)–(154) do not contain the Biot numbers $Bi_{l}$, l=1,2. Thus, in the initial stages of the frictional heating process, the effect of convective cooling on the temperature is negligible, and the temperature field is the same as in the case of the frictional heating of two semi-infinite bodies. By setting $\zeta =0^{\pm }$ in Equations (151)–(154), the dimensionless temperature rises in the friction surfaces of the layers in the initial period of the heating are written in the following form:

(155) $$\Theta^{*}(0^{+},\tau )≅\frac{2}{\left(1+\varepsilon \right)}\sqrt{\frac{\tau }{\pi }}-\frac{\lambda }{2\kappa }[1-e^{\kappa^{2}\tau }\mathrm{erfc}(\kappa \sqrt{\tau })],\;0\leq \tau <<1,$$

(156) $$\Theta^{*}(0^{-},\tau )≅\frac{2}{\left(1+\varepsilon \right)}\sqrt{\frac{\tau }{\pi }}+\frac{\lambda }{2\varepsilon \kappa }[1-e^{\kappa^{2}\tau }\mathrm{erfc}(\kappa \sqrt{\tau })],\;0\leq \tau <<1.$$

By further taking the limit Bi→∞ (κ→∞) in Equations (155) and (156), the known formulas for determining the maximum temperature under perfect thermal contact conditions are obtained [19]:

(157) $$\Theta^{*}(0^{+},\tau )=\Theta^{*}(0^{-},\tau )≅\frac{2\sqrt{\tau }}{\left(1+\varepsilon \right)},\mathrm{br}-\mathrm{to}-\mathrm{break}\;0\leq \tau <<1.$$

The verification of the proposed model was carried out by examining the convergence of the derived special (limiting) cases of the solution with the corresponding results reported in the literature. Another method of indirect verification involves comparing the results of numerical analyses with those obtained from appropriate finite element method (FEM) models. On the other hand, the exact analytical solutions serve as benchmarks for validating the results of numerical methods.

## 7. Numerical Analysis

Based on the proposed analytical model, a numerical analysis is conducted for a selected friction pair, where the first element (l=1) is a friction material with homogeneous properties, and the second layer (l=2) is made of a ceramic–metal FGM composite. The friction surface of the second element consists of pure ceramic ${\mathrm{ZrO}}_{2}$, and as the transverse distance from this surface increases, the volume fraction of the second component, the titanium alloy Ti-6Al-4V, continuously increases, forming the free (outer) surface of the FGM layer. Such an FGM is characterized by an increasing thermal conductivity coefficient as the distance from the working surface into the depth of the layer increases along its thickness. The thermal properties of the homogeneous friction material (l=1) and the FGM components (l=2) are presented in Table 1. With this selection of FGM components, the gradient parameter $\gamma^{*}=1.28$ is determined from relation (8).

The remaining input parameters assumed for the calculations are:

- Specific friction power $q_{0}=100,000\;{\mathrm{Wm}}^{-2}$;
- Contact conductance $h=100\;{\mathrm{Wm}}^{-2}K^{-1}$ [22], which gives a Biot number Bi=0.468 (9);
- Friction elements have the same thickness;
- Convective cooling intensity coefficients on the outer surfaces of the layers $h_{1}=h_{2}=100\;{\mathrm{Wm}}^{-2}K^{-1}$ [23]; thus $Bi_{1}=0.468$, and $Bi_{2}=0.133$ (9).

The above set of input data constitutes the reference case for further analysis.

**Figure 2:**
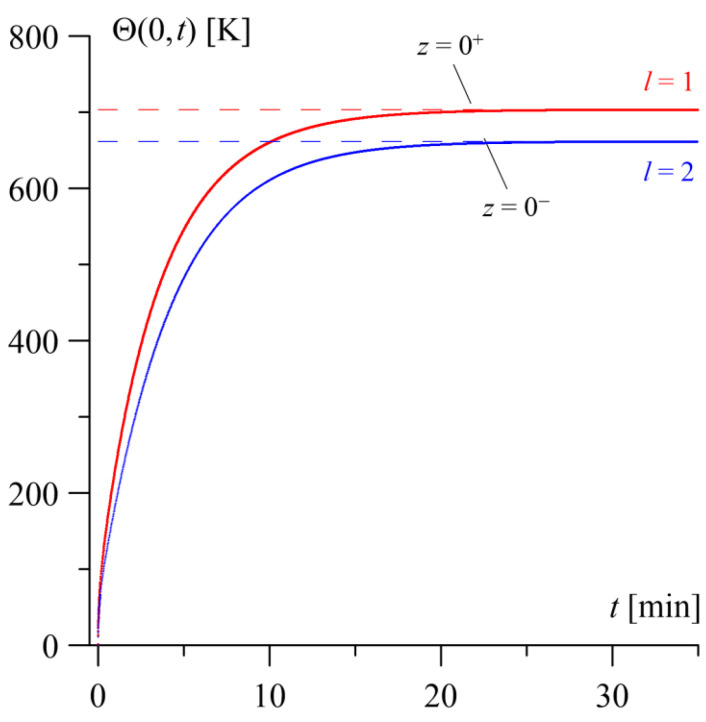
The evolutions of the temperature rise $\Theta_{l}(0^{\pm },t)$ (116) and (117) over time t (solid lines) on the contact surfaces of the homogeneous layer (l=1, red lines) and the FGM layer (l=2, blue lines), as well as the steady-state temperature levels $\Theta_{l,0}(0^{\pm })$ (110) (dashed lines).

The temperature evolutions $\Theta_{l}(0,t)$ (116) and (117) during heating over time t on the contact surfaces of the homogeneous and functionally graded (FGM) layers, along with their corresponding steady-state temperature levels (110), are presented in Figure 2. In the initial stage of sliding (t<10 min), a rapid increase in the surface temperature of both layers is observed. Over time, the rate of temperature rise gradually decreases until reaching the limit (maximum) values corresponding to the steady-state components of the solutions (110). This indicates that a steady state is reached, where the temperature level stabilizes.

On the surface of the homogeneous layer $z=0^{+}$, the temperature rise is more rapid, and the attained steady-state temperature $\Theta_{1,0}(0^{+})=703\;K$ is higher. Furthermore, the steady state is reached earlier at time $t_{1,0}=20\;\min$. In the case of the contact surface of the gradient layer $z=0^{-}$, more efficient heat dissipation into the depth of the element results in a lower maximum temperature of $\Theta_{2,0}(0^{-})=662\;K$, while the steady state is reached slightly later at time $t_{2,0}=21\;\min$.

**Figure 3:**
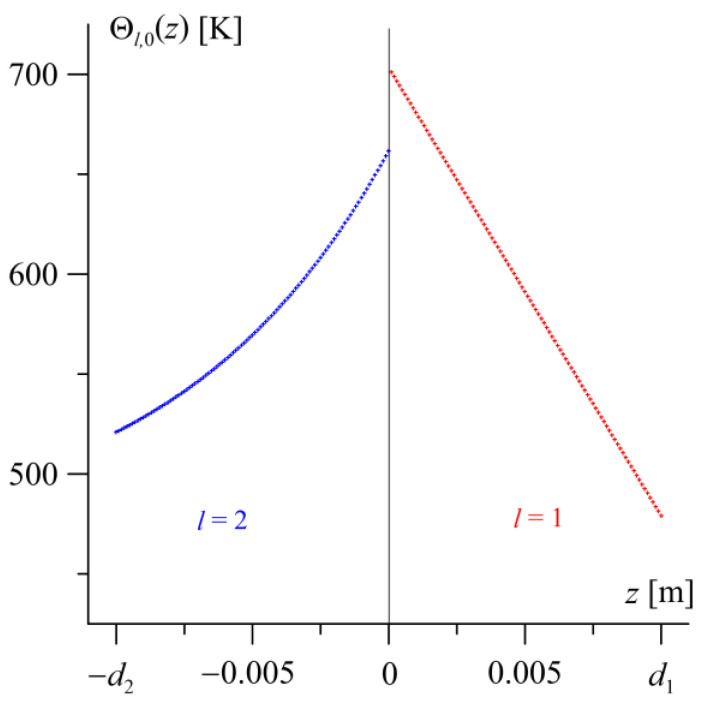
Steady-state temperature distribution $\Theta_{l,0}(z)$ along the thickness of the homogeneous (l=1, red lines) $0\leq z\leq d_{1}$ and FGM layers (l=2, blue lines) $-d_{2}\leq z\leq 0$.

After reaching the steady state, an analysis of the temperature distribution $\Theta_{l,0}(z)$ is conducted in the transverse direction of the friction pair elements $-d_{2}\leq z\leq 0$ (Figure 3). A visible jump in the steady-state temperature value between the contact surfaces of the friction pair elements is 41 K. Moving away from the friction surfaces into the depth of the elements, the temperature level decreases, and the temperature distribution in the layer with a constant thermal conductivity coefficient (l=1) is nearly linear. The temperature gradient $\Theta_{2,0}(0^{-})-\Theta_{2,0}(-d_{2})=141\;K$ along the thickness of the FGM layer is smaller than the gradient $\Theta_{1,0}(d_{1})-\Theta_{1,0}(0^{+})=224\;K$ in the homogeneous layer.

**Figure 4:**
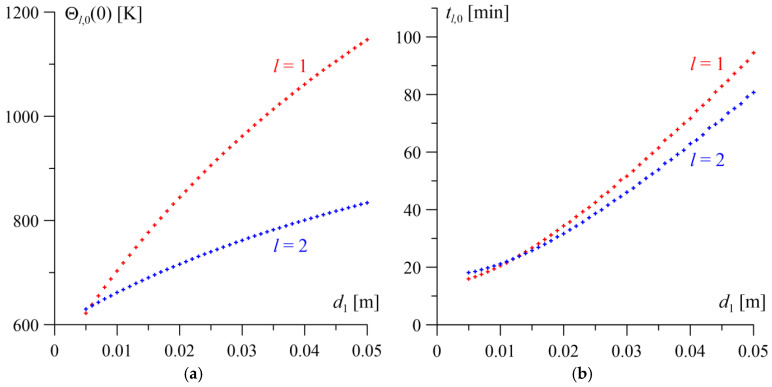
The influence of the change in the thickness of the homogeneous layer $d_{1}$ on the steady-state temperature $\Theta_{l,0}(0)$ (**a**) and the time to reach it $t_{l,0}$ (**b**) on the friction surfaces l=1, 2.

**Figure 5:**
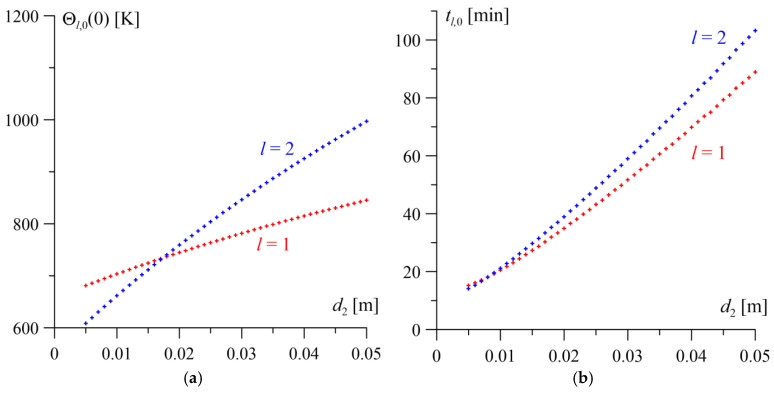
The effect of the change in the FGM layer thickness $d_{2}$ on the steady-state temperature $\Theta_{l,0}(0)$ (**a**) and the time to reach it $t_{l,0}$ (**b**) on the friction surfaces l=1, 2.

The results of the analysis concerning the impact of changing the thickness of one of the layers on the steady-state temperature level $\Theta_{l,0}(0)$ and the time to reach it $t_{l,0}$ on the friction surfaces of both elements are presented in Figure 4 and Figure 5. Increasing the thickness of either layer within the range $0.005\leq d_{l}\leq 0.05\;m$, l=1, 2, while maintaining the constant reference thickness of the other layer, leads to an increase in both the time to reach the steady state on the working surfaces of both elements and the steady-state temperature level. This effect is more pronounced for the element whose thickness is being varied.

Increasing the thickness of the homogeneous layer to $d_{1}=0.05\;m$ has a greater impact on the rise in the steady-state temperature $\Theta_{1,0}(0^{+})=1147\;K$ and the time to reach it on the surface of this layer (approximately 94 min), compared to the FGM layer, for which $\Theta_{2,0}(0^{-})=834\;K$ after 81 min (Figure 4a). Conversely, as the transverse dimension of the homogeneous layer decreases, the difference between the steady-state temperatures on the mating friction surfaces decreases until they equalize $\Theta_{1,0}=\Theta_{2,0}=638\;K$ at a thickness of $d_{1}=0.006\;m$. Changes in the transverse dimension of the gradient layer exert an analogous influence on the steady-state temperature distribution (Figure 5a). In this case, the equalization of the steady-state temperature levels of both layers $\Theta_{1,0}=\Theta_{2,0}=732\;K$ occurs at an FGM layer thickness of $d_{2}=0.017\;m$.

The changes in the time to reach the steady state on the surfaces of both layers as a function of the thickness of the homogeneous layer and the FGM layer are similar, both qualitatively and quantitatively (Figure 4b and Figure 5b). For the minimum considered thickness of one of the layers d=0.005 m, the steady-state temperature is reached after approximately 15–18 min of heating. A ten-fold increase in this dimension results in a significant extension of the temperature stabilization time to approximately 80–103 min.

**Figure 6:**
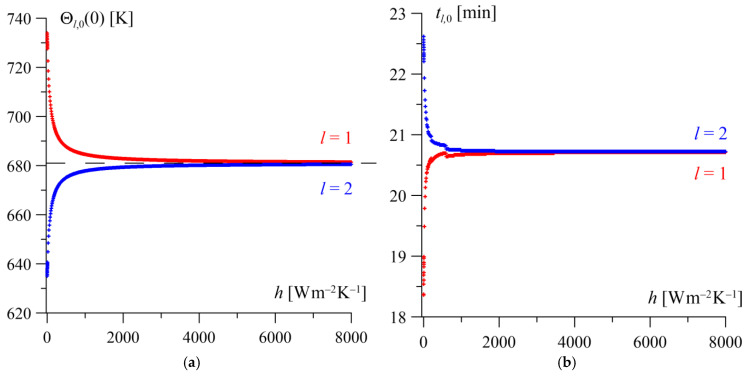
Changes in the steady-state temperature $\Theta_{l,0}(0)$ (**a**) and the time to reach it $t_{l,0}$ (**b**) on the friction surfaces of both elements l=1, 2, depending on the contact conductance h.

The effect of changes in the contact conductance h (the inverse of thermal contact resistance) in the range $0\leq h\leq 8000\;\;{\mathrm{Wm}}^{-2}K^{-1}$ is presented in Figure 6. It is worth noting that in engineering applications (e.g., in braking systems), the contact conductance coefficient h can take values on the order 10^4^ or even higher [22]. At a zero contact conductance coefficient (h=0), we obtain infinite thermal contact resistance, meaning that there is no heat flow between the elements of the friction pair at the interface of the mating surfaces. In this case, the highest jump in the steady-state surface temperature values is obtained: $\Theta_{1,0}=734\;K$ for the homogeneous layer and $\Theta_{2,0}=635\;K$ for the gradient layer (Figure 6a). Simultaneously, the largest discrepancy between the times to reach the steady state on the surfaces of the friction elements is observed, amounting to $t_{1,0}=18\;\min$ and $t_{2,0}=23\;\min$, respectively (Figure 6b).

As the contact conductance h increases, both profiles of the steady-state temperature changes for both layers tend toward a constant limit value (horizontal asymptote) equal to $\Theta_{l,0}=681\;K$, l=1, 2. The surface temperature level of the homogeneous layer decreases, while it increases on the surface of the gradient layer. The most rapid changes in both the steady-state temperatures and the time to reach the steady state occur in the range of low h values. In turn, at high values of contact conductance $h\geq 7200\;{\mathrm{Wm}}^{-2}K^{-1}$, the steady-state temperatures of both friction surfaces equalize (with an accuracy of ± 1 K) and are reached after the same time $t_{l,0}=20.75\;\min$ (Figure 6a).

**Figure 7:**
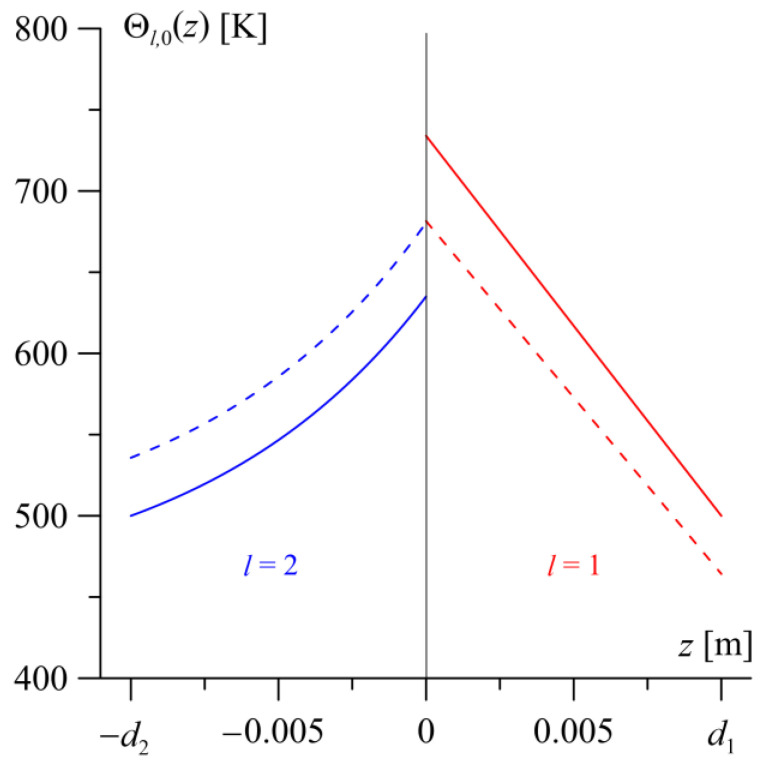
Steady-state temperature distributions $\Theta_{l,0}(z)$ along the thickness of the homogeneous layer (l=1, red lines) $0\leq z\leq d_{1}$ and the FGM layer (l=2, blue lines) $-d_{2}\leq z\leq 0$ under conditions close to perfect thermal contact ($h=7200\;{\mathrm{Wm}}^{-2}K^{-1}$, dashed lines) and in the absence of heat exchange between the contact surfaces of the layers (h=0, solid lines).

The steady-state temperature distributions in the transverse direction of the layers $-d_{2}\leq z\leq d_{1}$ at different values of the contact conductance coefficient h are illustrated in Figure 7. The solid lines represent the temperature distribution assuming no heat exchange between the contact surfaces (h=0). A significant jump in temperature value equal to 99 K is visible between the friction surfaces of the layers (z=0). In contrast, under conditions close to perfect thermal contact ($h>7200\;{\mathrm{Wm}}^{-2}K^{-1}$, Bi>33.7), we see the continuity of the temperature distribution at the interface (dashed lines in Figure 7).

**Figure 8:**
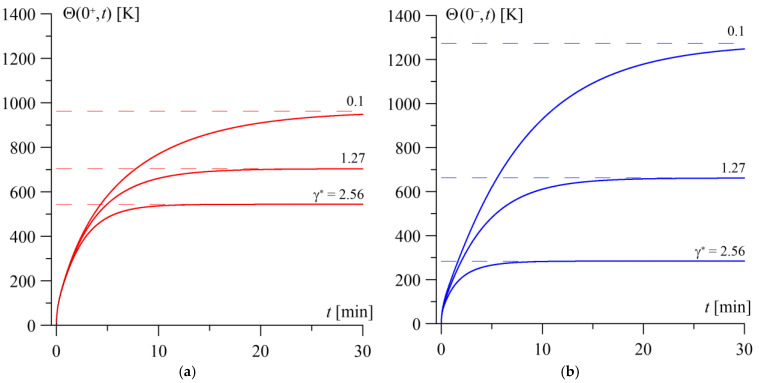
The evolutions of the temperature rise $\Theta_{l}(0,t)$ over heating time t at various values of the gradient parameter $\gamma^{*}$ on the contact surfaces of the homogeneous layer (l=1) (**a**) and the FGM layer (l=2) (**b**).

**Figure 9:**
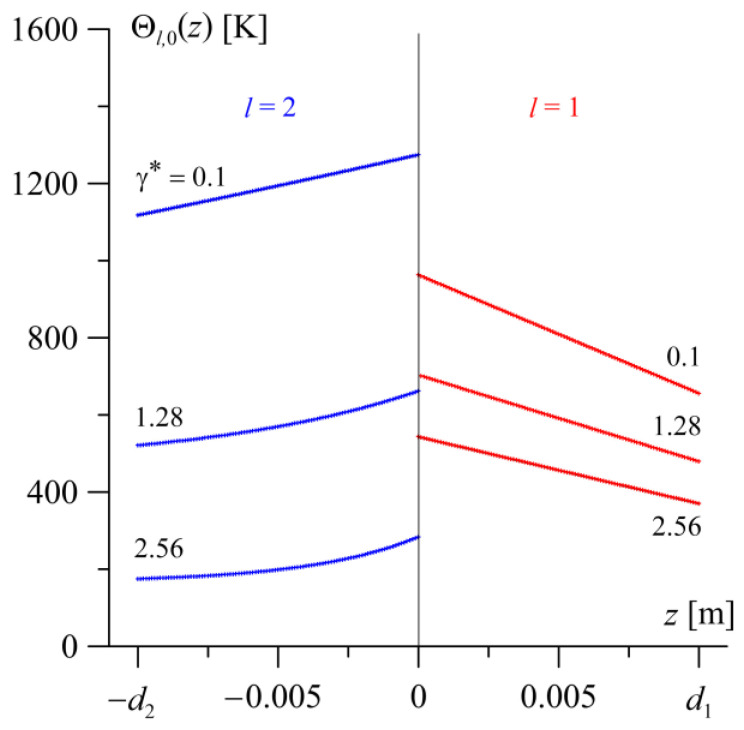
Steady-state temperature distributions $\Theta_{l,0}(z)$ along the thickness of the homogeneous layer (l=1, red lines) $0\leq z\leq d_{1}$ and the FGM layer (l=2, blue lines) $-d_{2}\leq z\leq 0$ at various values of the gradient parameter $\gamma^{*}$.

The influence of the gradient parameter of the second layer material (l=2) on the temperature evolution on the friction surfaces of the elements during sliding (Figure 8) and the temperature distribution along the thickness of the layers after reaching the steady state (Figure 9) was investigated. Changing the value of the gradient parameter exerts a significantly greater influence on the temperature of the FGM layer than on the counter-element (the homogeneous layer).

In the case of a two-component FGM (ZrO_2_–Ti-6Al-4V), decreasing the value of the gradient parameter $\gamma^{*}$ relative to the reference value $\gamma^{*}=1.28$ can be achieved by increasing the volume fraction of the ceramic component in the material. At small values of the gradient parameter, we obtain a composite consisting primarily of ${\mathrm{ZrO}}_{2}$ ceramic, with a small admixture of a titanium alloy on the outer side of the layer. By increasing the volume fraction of the ceramic component to approximately 95% of the FGM, we would obtain a gradient parameter value of $\gamma^{*}=0.1$. In this case, the steady-state temperature levels reached on the working surfaces of the elements during friction are at their highest $\Theta_{1,0}=963\;K$ and $\Theta_{2,0}=1274\;K$, and the time to reach the steady state is the longest (Figure 8).

The temperature distribution in such an almost entirely ceramic ($\gamma^{*}=0.1$) layer is similar to the distribution in a homogeneous material (l=1)—nearly linear (Figure 9). As the FGM gradient increases, the system temperature decreases, and the temperature distribution in the transverse direction becomes increasingly non-linear; specifically, the temperature drops more rapidly within the depth of the layer. This indicates the improved cooling of the layer at the outer surface $z=-d_{2}$ as a result of convective heat exchange with the environment.

Increasing the gradient parameter relative to the reference value can be achieved, for example, by replacing the metallic component of the FGM with a material of higher thermal conductivity than the titanium alloy Ti-6Al-4V. By replacing this material with steel, which has a thermal conductivity coefficient more than three times higher, we obtain an FGM with a doubled gradient parameter $\gamma^{*}=2.56$. In this case, a friction pair is obtained, in which the steady-state surface temperatures are the lowest, and the steady state is reached the earliest.

**Figure 10:**
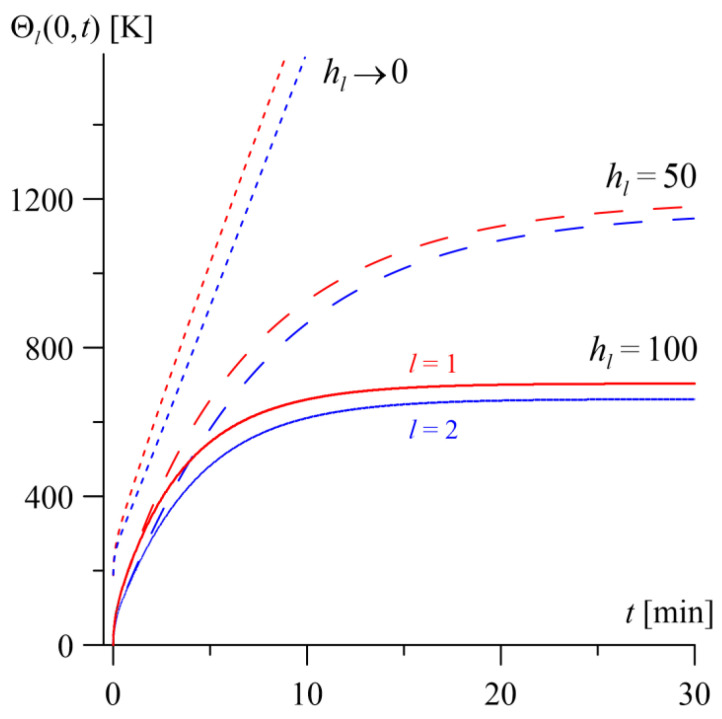
Temperature evolutions $\Theta_{l}(0,t)$ over time t on the contact surfaces of the homogeneous layer (l=1, red lines) and the gradient layer (l=2, blue lines), for different intensities of convective cooling on the outer surfaces l=1, 2: $h_{l}=100\;{\mathrm{Wm}}^{-2}K^{-1}$ (solid lines); $50\;{\mathrm{Wm}}^{-2}K^{-1}$ (dashed lines) and $h_{l}\to 0$ (dotted lines).

The temperature profiles on the friction surfaces of the layers during 30 min of frictional heating for three different values of the convective heat exchange coefficient $h_{l}$ on the outer surfaces of the layers l=1, 2 are presented in Figure 10. For typical braking processes with natural (unforced) convective cooling, the value of this coefficient is usually assumed to be up to $h_{l}=100\;{\mathrm{Wm}}^{-2}K^{-1}$ [23], while in systems with a ventilated brake disk, it can reach up to $300\;{\mathrm{Wm}}^{-2}K^{-1}$ [24,25].

At a value of $h_{l}=50\;{\mathrm{Wm}}^{-2}K^{-1}$ (dashed lines in Figure 10), the surface temperature of both layers rises to a significantly higher level, and the rise time (time to reach the steady state) is extended compared to the reference case $h_{l}=100\;{\mathrm{Wm}}^{-2}K^{-1}$ (solid lines in Figure 10), which corresponds to twice the intensity of convective cooling.

In contrast, neglecting the convective heat exchange with the environment on the outer surfaces of the layers $h_{l}\to 0$ (dotted lines in Figure 10) leads to a very rapid temperature increase that is nearly linear in nature. In this case, a practically unlimited increase in the steady-state temperature is observed, meaning that the system under consideration does not reach a steady state.

The influence of the convective heat exchange coefficient on the outer surfaces of the layers on the surface temperature level during sliding is qualitatively the same for both the homogeneous layer (l=1) and the gradient layer (l=2).

## 8. Conclusions

In this study, the heat transfer process during the uniform sliding of two layers was considered, where one is made of a functionally graded material (FGM) with exponentially varying thermal conductivity, and the other is a homogeneous material. To develop the model for this process, an appropriate thermal friction problem was formulated, accounting for thermal contact conductance and convective heat exchange with the environment on the free surfaces of the layers. An exact solution, including special cases of this problem, was obtained using analytical methods. Based on the developed model, the thermal behavior of a friction pair consisting of a ceramic–metallic FGM layer (ZrO_2_–Ti-6Al-4V) coupled with a homogeneous friction material was investigated. The obtained results allowed for the formulation of the following conclusions:

- The process of the frictional heating of layers at constant friction power is characterized by a monotonic increase in the temperature until the system stabilizes in a stationary temperature regime.
- In the absence of convective heat transfer to the ambient environment, the system does not reach a steady state. Conversely, increasing the convective cooling intensity coefficient on the outer surfaces of the layers leads to a reduction in the contact temperature level and shortens the time required to reach the stationary temperature.
- An increase in the gradient parameter of the FGM layer results in a significant reduction in the surface temperature of both friction pair elements (this effect is much more pronounced for the gradient layer) and shortens the system’s stabilization time. Additionally, it improves the convective heat dissipation process through exchange with the surrounding environment on the outer surface of the FGM layer. This results in a more uniform temperature distribution than in a homogeneous layer, which translates to lower thermal stress values and a reduced risk of crack initiation [26,27].
- Increasing the thickness of either layer (within the range of 5–50 mm) causes an extension of the time to reach the steady state and an increase in the stationary temperature on the working surfaces. The effect is stronger for the layer whose thickness is being varied.
- At high contact conductance h, the conditions at the interface approach those of perfect thermal contact, i.e., the temperature levels on the mating friction surfaces equalize, as do the times required to reach the steady state. Decreasing the heat exchange between the friction surfaces results in an increased temperature jump across the interface.
- The temperature jump between the mating surfaces of the friction pair elements is primarily determined by the thermal contact conditions, the thickness of the friction elements and the material gradient parameter of the FGM.

The conducted analysis allowed for determining the influence of the FGM element’s gradient parameter, the intensity of convective heat exchange with the environment, contact conductance, and the thickness of the friction elements on the evolution of surface temperature, temperature distribution along the thickness of the layers, and the time to reach a steady state. The results obtained revealed significant differences in the thermal behavior of the homogeneous and gradient layers. Utilizing an FGM with an appropriate gradation leads to lowering the maximum surface temperatures, reducing temperature gradients into the depth of the element and shortening the thermal stabilization time. The findings also confirmed the crucial role of heat exchange between the layers at the contact surface and the thickness of the friction pair elements on the temperature jump at the interface and the time required to reach the steady state.

The developed model allows for the assessment of temperature in friction systems and for evaluating the influence of key design parameters in applications involving functionally graded materials. Such analytical solutions are particularly valuable for parametric analyses of various tribological components operating under sliding contact conditions, including braking systems and clutch assemblies [28,29], although many friction processes in such systems are transient in nature and not stationary [30]. However, the developed solution for constant-specific friction power constitute the basis for the analytical determination of appropriate non-stationary solutions, which can be conducted using Duhamel’s theorem [31,32].

## Figures and Tables

**Table 1:** Thermophysical properties of materials [20,21].

Material	Element	$\mathrm{Thermal}\;\mathrm{Conductivity}\;K_{l}\;{[\mathrm{Wm}}^{-1}K^{-1}]$	$\mathrm{Specific}\;\mathrm{Heat}\;\mathrm{Capacity}\;C_{l}\;[J\;{\mathrm{kg}}^{-1}K^{-1}]$	$\mathrm{Density}\;\rho_{l}\;[\mathrm{kg}\;m^{-3}]$
${\mathrm{ZrO}}_{2}$	*l* = 2	2.09	456.7	4420
Ti-6Al-4V		7.5	537	5331
friction composite	*l* = 1	2.137	986	1787

## Data Availability

The original contributions presented in this study are included in the article. Further inquiries can be directed to the corresponding author.

## Nomenclature

Bi	contact Biot number, dimensionless
$Bi_{l}$	convective Biot numbers, dimensionless
$C_{l}$	specific heat, J kg^−1^ K^−1^
$d_{l}$	thickness of the layer, m
$d^{*}$	relative thickness of the layers, dimensionless
f	coefficient of friction, dimensionless
h	thermal contact conductivity, W m^−2^ K^−1^
$h_{l}$	convective heat transfer coefficients, W m^−2^ K^−1^
$I_{n}(\cdot )$	modified Bessel functions of the nth order of the first kind
$J_{n}(\cdot )$	Bessel functions of the nth order of the first kind
$K_{n}(\cdot )$	modified Bessel functions of the nth order of the second kind
$k_{l}$	thermal diffusivity of the layer, m^2^ s^−1^
$K_{l}$	thermal conductivity of the layer, W m^−1^ K^−1^
$K_{l,m}$	thermal conductivity of the mth component of the FGM layer, W m^−1^ K^−1^
$q_{0}$	specific friction power, W m^−2^
p	parameter of the Laplace transform, dimensionless
$p_{0}$	pressure, Pa
t	time, s
$t_{l,0}$	time to reach the steady state at the friction surface of the layer, s
T	temperature, K
$T_{0}$	initial temperature, K
$V_{0}$	sliding velocity, m s^−1^
$Y_{n}(\cdot )$	Bessel functions of the *n*th order of the second kind
z	axial coordinate, m
$\gamma^{*}$	gradient parameter of the FGM, dimensionless
ε	relative thermal activity coefficient, dimensionless
Θ	temperature rise, K
$\Theta^{*}$	dimensionless temperature rise,
$\Theta_{0}$	temperature scaling coefficient, K
$\Theta_{l,0}$	steady-state temperature level, K
$\rho_{l}$	density, kg m^−3^
τ	Fourier number, dimensionless
ζ	axial coordinate, dimensionless
